# HIF-2*α* and Oct4 have synergistic effects on survival and myocardial repair of very small embryonic-like mesenchymal stem cells in infarcted hearts

**DOI:** 10.1038/cddis.2016.480

**Published:** 2017-01-12

**Authors:** Shaoheng Zhang, Lan Zhao, Jiahong Wang, Nannan Chen, Jian Yan, Xin Pan

**Affiliations:** 1 Department of Cardiology, the Third Affiliated Hospital of Southern Medical University, 183 West Zhongshan Road, Tianhe District, Guangzhou 510630, China; 2 Department of Cardiology, Dahua Hospital, 901 Laohumin Rd, Xuhui District, Shanghai 200237, China; 3 Department of Cardiology, Yangpu Hospital, Tongji Univercity School of Medicine, 450 Tengyue Rd, Shanghai 200090, China; 4 Central Laboratory, Yangpu Hospital, Tongji Univercity School of Medicine, 450 Tengyue Rd, Shanghai 200090, China

## Abstract

Poor cell survival and limited functional benefits have restricted mesenchymal stem cell (MSC) efficacy for treating myocardial infarction (MI), suggesting that a better understanding of stem cell biology is needed. The transcription factor HIF-2*α* is an essential regulator of the transcriptional response to hypoxia, which can interact with embryonic stem cells (ESCs) transcription factor Oct4 and modulate its signaling. Here, we obtained very small embryonic-like mesenchymal stem cells (vselMSCs) from MI patients, which possessed the very small embryonic-like stem cells' (VSELs) morphology as well as ESCs' pluripotency. Using microarray analysis, we compared HIF-2*α*-regulated gene profiles in vselMSCs with ESC profiles and determined that HIF-2*α* coexpressed Oct4 in vselMSCs similarly to ESCs. However, this coexpression was absent in unpurified MSCs (uMSCs). Under hypoxic condition, vselMSCs exhibited stronger survival, proliferation and differentiation than uMSCs. Transplantation of vselMSCs caused greater improvement in cardiac function and heart remodeling in the infarcted rats. We further demonstrated that HIF-2*α* and Oct4 jointly regulate their relative downstream gene expressions, including Bcl2 and Survivin; the important pluripotent markers Nanog, Klf4, and Sox2; and Ang-1, bFGF, and VEGF, promoting angiogenesis and engraftment. Importantly, these effects were generally magnified by upregulation of HIF-2*α* and Oct4 induced by HIF-2*α* or Oct4 overexpression, and the greatest improvements were elicited after co-overexpressing HIF-2*α* and Oct4; overexpressing one transcription factor while silencing the other canceled this increase, and HIF-2*α* or Oct4 silencing abolished these effects. Together, these findings demonstrated that HIF-2*α* in vselMSCs cooperated with Oct4 in survival and function. The identification of the cooperation between HIF-2*α* and Oct4 will lead to deeper characterization of the downstream targets of this interaction in vselMSCs and will have novel pathophysiological implications for the repair of infarcted myocardium.

Mesenchymal stem cells (MSCs) are multipotent, easily obtainable, have low immunogenicity, and secrete angiogenic factors that promote cardiac repair after myocardial infarction (MI).^1^ However, the therapeutic potency of transplanted MSCs appears to be limited by low rates of engraftment, survival, and differentiation:^2^ the percentage of transplanted MSCs in hearts declined from 34–80% immediately after administration to just 0.3–3.5% after 6 weeks;^3^ in a swine model of chronic ischemic cardiomyopathy, 10% of MSCs participated in coronary angiogenesis, and 14% differentiated into cardiomyocytes.^4^ Accordingly, researchers have developed methods to improve the survival and effectiveness of transplanted cells by genetically manipulating the expression of proteins that regulate antioxidant resistance, vascular growth and the apoptotic response to ischemic injury.^5, 6^ One problem that remains is whether the persistent expression of foreign proteins could lead to malignant transformation or transplantation failure, supporting the hypothesis that new strategies for exploring the endogenous cytoprotection and survival advantage to improve the effect of stem cell therapy would be more favorable. The primary transcriptional regulators of both cellular and systemic hypoxic adaptation in mammals are hypoxia-inducible factors (HIFs). HIFs regulate the expression of many genes involved in the survival and effects of transplanted cells, but which remains elusive.^7^ Most of our current knowledge about these transcription factors is based on studies of HIF-1*α* and, to a lesser degree, HIF-2*α*. Forristal *et al.* found that silencing of HIF-2*α* resulted in a significant decrease in human embryonic stem cell (hESC) proliferation and the protein expressions of Oct4, SOX2 and NANOG.^8^ Covello *et al.* showed that HIF-2*α* can regulate ESCs function and/or differentiation through activation of Oct-4,^9^ suggesting that HIFs in combination with Oct4 are essential for ESC survival. How the relation between Oct4 and HIFs by ischemia leads to MSC death or survival, and the attendant transcriptional activity, is unknown.

MSCs produce a variety of cytokines, such as vascular growth factor (VEGF), basic fibroblast growth factor (bFGF), and angiopoietin-1 (Ang-1), which directly promote cell survival and have beneficial effects on myocardial repair following MI.^10, 11^ In some cases, MSC sorting based on markers appears to enrich subpopulations of MSCs with differing paracrine activity.^12^

This led to our development of a population of vselMSCs using hypoxic culture and ESC culture conditions in combination with our previously described methods^11^ from the patients with acute MI. The present study was designed to gain insights into the autologous expression of HIFs, Oct4, anti-apoptotic factors, and angiogenic cytokines in vselMSCs under hypoxic conditions. We then demonstrated the functional cooperation between HIFs and Oct4 in myocardial repair induced by autologous vselMSC therapy combined with HIF-2*α* or Oct4 overexpression.

## Results

### Comparison of the VSELs in circulating blood MNCs

**Figure 1:**
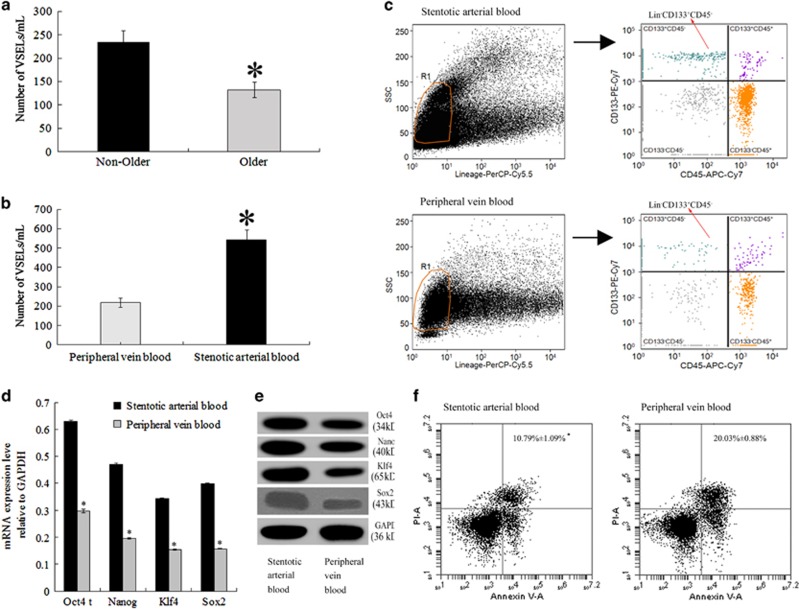
VSEL properties. (**a**) Age-dependent frequency of VSEL cell subsets expressing CD133^+^Lin^−^CD45^−^ into the PB. Two groups of patients with STEMI were designated according to age: Non-Older (20–60 years), Older (>60–75 years). The frequency of CD133^+^Lin^−^CD45^−^ cell subsets was calculated per ml PB. *P<0.05 for comparison between the groups (*n*=10 per group). (**b**) Bar graphs showing the absolute numbers of circulating CD133^+^Lin^−^CD45^−^ cells in the peripheral vein and stenotic coronary artery of patients with STEMI; there was peak mobilization early in the patients. *P<0.05 for comparison between the stenotic arterial blood and the peripheral vein blood (*n*=10 per group). (**c**) depicts cryptograms of the MNC population and gating strategy starting from Lineage *versus* side scatter (SSC). Cells were visualized by dot plot showing Lineage-PerCP-Cy5.5 *versus* SSC characteristics, which are related to Lineage negative (Lin^−^) and granularity/complexity, respectively (left). Objects from gate R1 were further analyzed for CD133 and CD45 expression, and only CD133^+^CD45^−^ events were selected. The population from gate R1 was subsequently sorted based on CD45 marker expression into Lin^−^/CD133^+^/CD45^−^ VSELs, which are visualized in the histogram (right). (**d**) qRT-PCR evaluation of Oct4, Nanog, Klf4, and Sox2 mRNA levels. *P<0.05 for comparison between SB and PB (*n*=10 per group). (**e**) Representative immunoblot electrophoresis showing Oct4, Nanog, Klf4, and Sox2 protein levels in VSELs from SB and PB. (**f**) Apoptotic cell death was assessed by annexin V-PI staining. *P<0.05 for comparison between SB and PB (*n*=10 per group)

Some data confirm that VSEL mobilization induced by acute MI differ according to age.^13^ Our study shows the same change trend: comparing with the enrolled patients with the older patients, we observed a statistically significant difference in VSEL numbers in the peripheral vein blood (PB) between the two groups (Figure 1a). The data suggested that patients aged 20–60 years had stronger mobilization of VSELs into the PB after AMI. Accordingly, we selected this age group for subsequent study. The number of circulating VSELs was significantly higher in the stenotic coronary arterial blood (SB) than in that from PB (Figure 1b). The Lin^−^/CD133^+^/CD45^−^ population number from gate R1 was greater in the SB than in the PB (Figure 1c). The qRT-PCR and immunoblotting showed that the SB VSELs expressed higher levels of Oct4, Nanog, Klf4, and Sox2 mRNA and protein than the PB (Figures 1d and e). Compared with the SB VSELs, 4-h simulated hypoxia induced less SB VSEL apoptotic cell death (Figure 1f). These data suggest that the SB contains a larger pool of anti-apoptotic VSELs as compared to PB. Therefore, we chose blood MNCs from the affected coronary artery to isolate and purify VSELs.

### vselMSC unique characteristics

**Figure 2:**
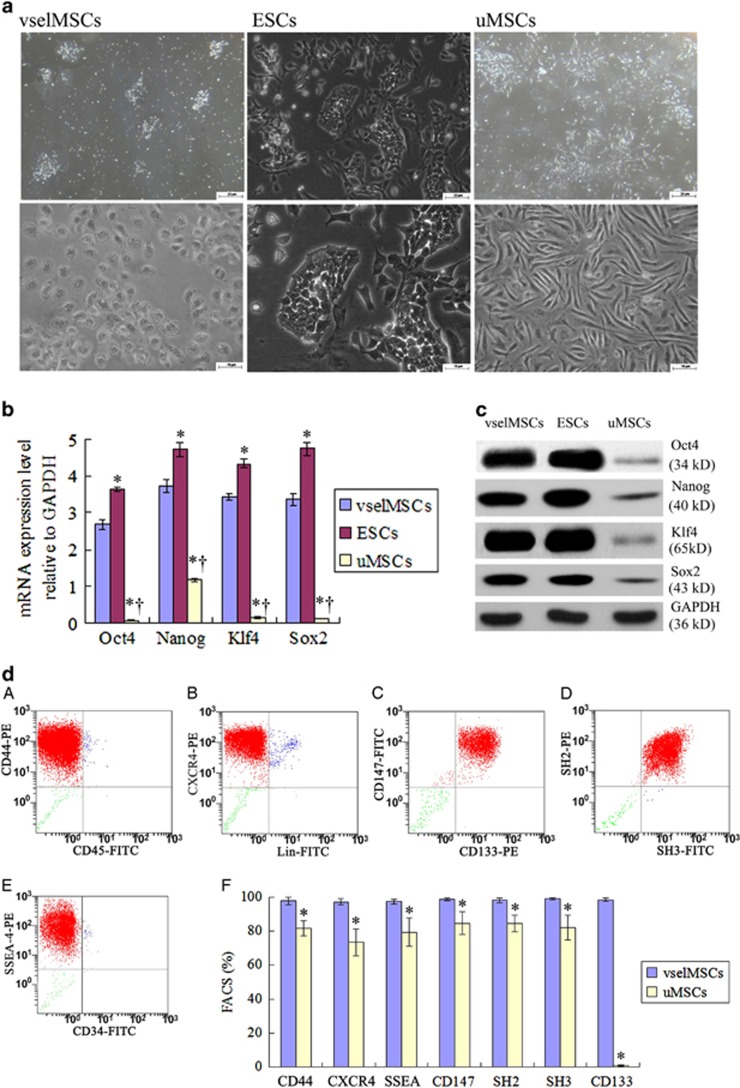

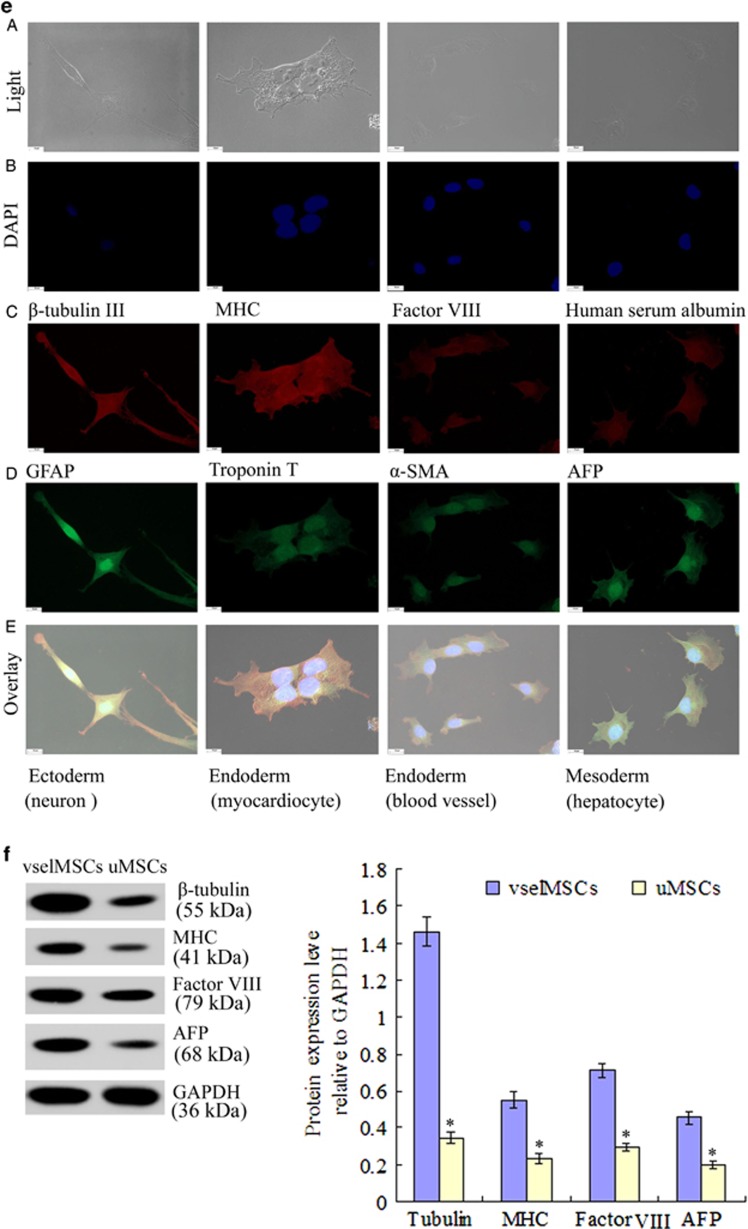
Characterization of vselMSCs. MSCs were collected from the affected coronary artery and filtered to obtain a population of vselMSCs. (**a**) vselMSCs, ESCs, and uMSCs were cultured in ESC medium and MSC medium, and compared morphologically under a bright-field microscope (upper panels: 10 × magnification, bars=25 *μ*m; lower panels: × 20 magnification, bars=10 *μ*m). (**b**) mRNA levels of the pluripotency markers Nanog, Klf4, Sox2, and Oct4 evaluated in vselMSCs, ESCs and uMSCs via qRT-PCR and normalized to GAPDH mRNA levels. *P<0.05 *versus* vselMSCs, ^†^*P*<0.05 *versus* ESCs (*n*=10 per group). (**c**) Oct4, Nanog, Klf4, and Sox2, protein levels in vselMSCs, ESCs and uMSCs compared via western blotting; GAPDH levels were used as the protein loading control. (**d**) The proportions of vselMSCs that expressed MSC (SH2 and SH3), ESC (SSEA), and VSEL (CD133 and CXCR4) markers, the matrix receptor CD44, and the endothelial marker CD147 were determined via flow cytometry (**A**–**E**). (**F**) FACS analysis of CD44, CXCR4, SSEA, CD147, SH2, SH3, and CD133 expression levels between vselMSCs and uMSCs. *P<0.05 *versus* vselMSCs (*n*=10 per group). (**e**) vselMSCs were induced to differentiate into cells from all three developmental germ layers (ectoderm: column 1; endoderm: columns 2–3; and mesoderm: column 4). The differentiated cells were examined morphologically (**A**) and via immunofluorescence (**B**–**E**) for the expression of ectodermal cell markers (i.e., the neuron-specific proteins *β*-tubulin III and glial fibrillary acidic protein [GFAP]), endodermal cell markers (i.e., the cardiomyocyte-specific markers troponin T and myosin heavy chain [MHC], and the vascular-cell specific proteins factor VIII and *α*-sarcomeric actin [*α* −SMA]), and mesodermal cell markers (i.e. the hepatic-cell markers serum albumin and alpha-fetoprotein [AFP]). The nuclei were stained with DAPI (blue), and the cytoplasm was stained red with anti-*β*-tubulin III, MHC anti-factor VIII, or serum albumin, and green with GFAP, troponin T, *α*-SMA, or AFP, respectively. Bars=10 *μ*m. (**f**) Representative immunoblot electrophoresis and subsequent quantification showing *β*-tubulin III, MHC, factor VIII, and AFP protein levels. *P<0.05 *versus* vselMSCs (*n*=10 per group)

Morphologically, cells from smaller (<200 *μ*m) colonies more closely resembled ESCs, with a rounded shape, large nuclei and scant cytoplasm (Figure 2a). Figure 2a also shows a side-by-side comparison of vselMSCs (3–4 *μ*m in diameter) with uMSCs (20–25 *μ*m in diameter). The mRNA levels of the pluripotency markers *Nanog*, *Klf4*, *Sox2*, and (especially) *Oct4* were significantly higher in vselMSCs than in uMSCs (Figure 2b). The protein levels correlated with the mRNA measurements (Figure 2c). More than 97% of vselMSCs expressed well-established markers for MSCs (SH2 and SH3), ESCs (SSEA), and VSELs (CD133 and CXCR4), as well as CD44 (matrix receptor), and CD147 (endothelial marker; Figures 2d(A–E)), and the expression levels of these markers were higher in the vselMSCs than in the uMSCs (Figures 2dF).

Next, we performed directed differentiation toward the ectoderm, endoderm, and mesoderm by growth factor supplementation and growth on defined matrices.^14, 15^ After induction, light microscopy showed characteristic morphologies of nerve cells, myocardiocytes, blood vascular cells, and hepatocytes (Figures 2eA). Immunofluorescence showed that the vselMSCs positively coexpressed the neuron marker *β*-tubulin III, the astrocyte-specific protein GFAP, myocardiocyte markers, troponin T and MHC, blood vascular markers, factor VIII and *α*-SMA, hepatocyte marker proteins, human serum albumin, and AFP (Figures 2eB–E). Western blotting revealed higher *β*-tubulin III, MHC, factor VIII, and AFP expression in vselMSCs as compared with uMSCs (Figure 2f).

### Oct4 interactome in vselMSCs includes HIF-2*α* protein

**Figure 3:**
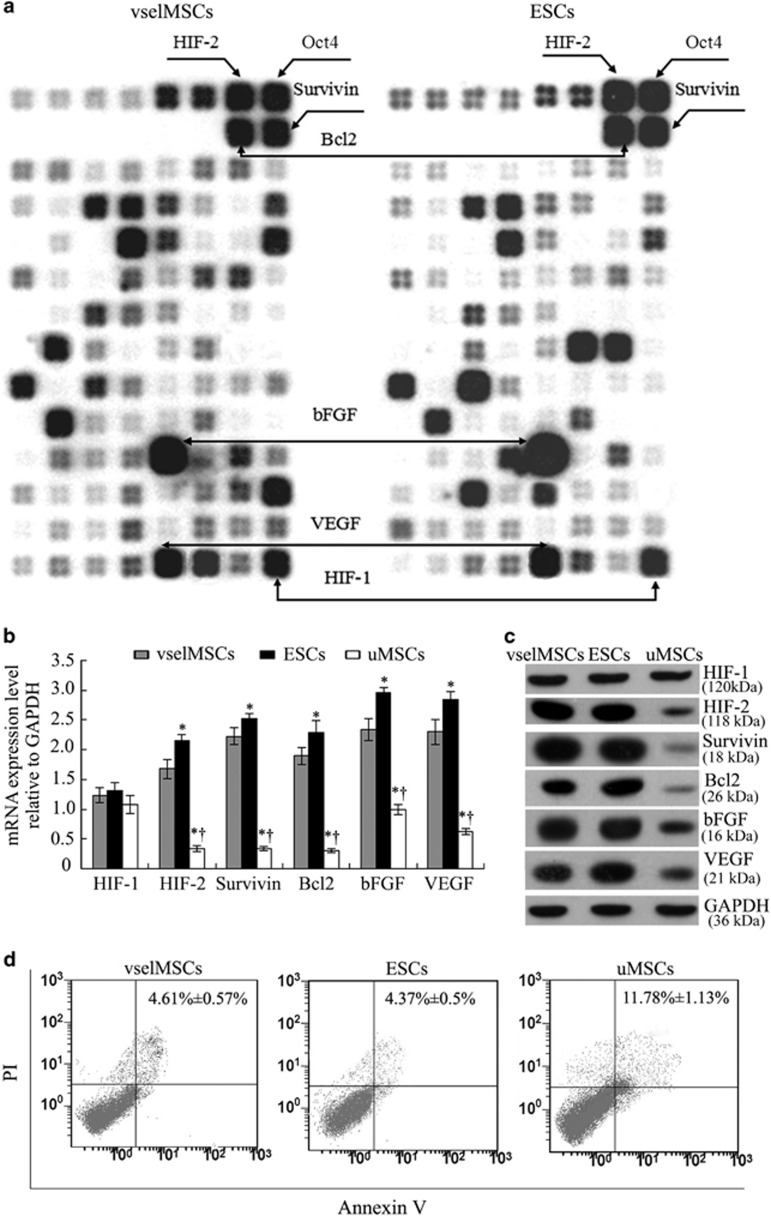
Identification of HIF-2α interacting proteins in vselMSCs. (**a**) Patterns of anti-apoptotic gene expression evaluated via gene expression array analysis in vselMSCs and ESCs cultured under normoxic conditions. (**b**) mRNA (qRT-PCR) and (**c**) protein levels (western blotting) of HIF-1 and HIF-2, and of four genes that are regulated by HIF (survivin, Bcl2, bFGF, and VEGF), evaluated in normoxia-cultured vselMSCs, ESCs and uMSCs. *P<0.05 *versus* vselMSCs, ^†^*P*<0.05 *versus* ESCs (*n*=10 per group). (**d**) Apoptosis (annexin V) and cell death (propidium iodide (PI)) were evaluated in normoxia-cultured vselMSCs, ESCs, and uMSCs via flow cytometry

As Oct4 acts as a stem cell marker,^16^ we evaluated the presence of HIF motifs around the Oct4-occupied regions in the data from vselMSCs and ESC. There were 16 genes that were expressed by more than 2- fold relative to GAPDH in vselMSCs, and there were seven shared genes that were uniquely common to vselMSCs and hESCs data sets (HIF-1, HIF-2, Oct4, bFGF, VEGF, Survivin, and Bcl2). HIF-2*α* motifs were enriched adjacent to the Oct4 motifs in vselMSCs, and were also detectable in hESCs (Figure 3a). The mRNA and protein expression levels of HIF-2, bFGF, VEGF, Survivin and Bcl2 were significantly higher in vselMSCs than in uMSCs, and slightly lower than in ESCs (Figures 3b and c), while HIF-1 expression in all three cell types was similar. The death rate was similar between vselMSCs and ESCs, and significantly lower in vselMSCs than in uMSCs (Figure 3d). The expression of the HIF-2 protein was negatively correlated with the apoptotic cell death ratio of vselMSCs, as assessed by FACS (*r*=−0.951, *P*<0.01), and positively correlated with the protein expressions of Oct4, Bcl2, Survivin, bFGF, and VEGF (*r*=0.929, 0.842, 0.930, 0.902, and 0.871, respectively; *P*<0.01 for all comparisons), showing the most significant positive correlation of Oct4 protein expression with HIF-2*α* expression among these interactors.

### HIF-2*α* interacts with Oct4, and both are essential for vselMSCs growth

**Figure 4:**
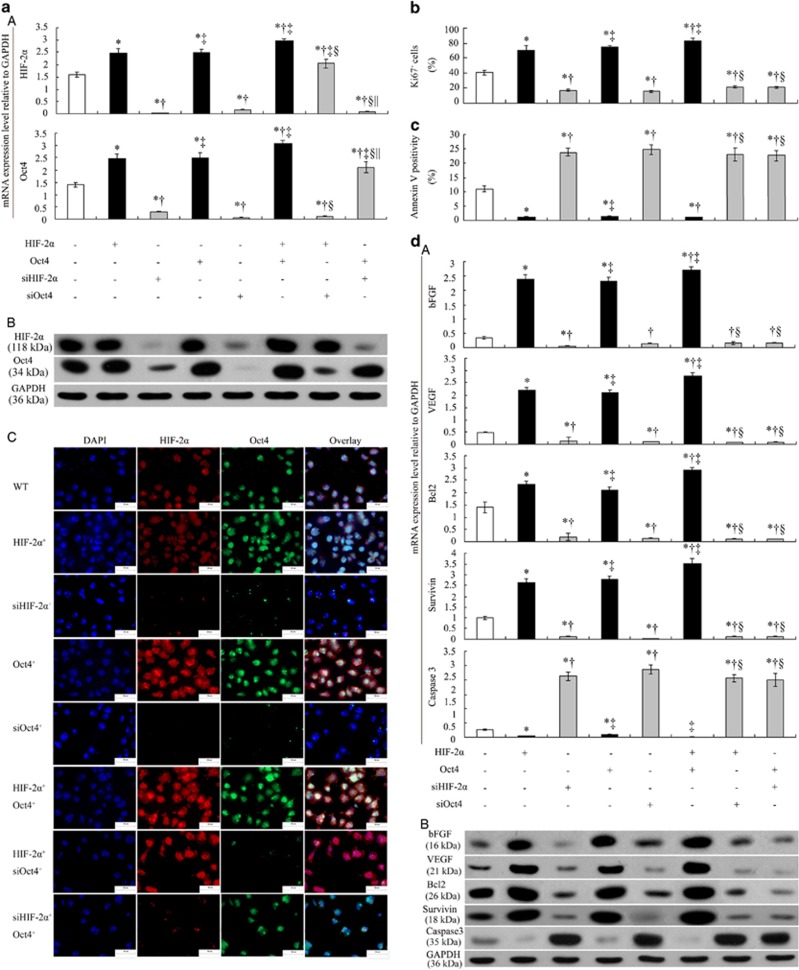
HIF-2α and Oct4 promote vselMSC growth*.* vselMSCs were transfected with vectors encoding HIF-2*α*, HIF-2*α* siRNA (siHIF-2*α*), Oct4, or Oct4 siRNA (siOct4) and cultured under hypoxic conditions. (**a**) qRT-PCR (A) and western blot (B) analysis of HIF-2*α* and Oct4 mRNA and protein expression, respectively, revealing that the two genes were significantly increased in vselMSCs overexpressing HIF-2*α* or Oct4 as compared with control vselMSCs and that expression was highest in cells co-overexpressing HIF-2*α* and Oct4. Silencing HIF-2*α* or Oct4 significantly reduced expression of the corresponding mRNA and protein. Overexpressing one transcription factor while silencing the other significantly increased the former and decreased the latter. *P<0.05 *versus* vehicle, ^†^*P*<0.05 *versus* HIF-2*α* or Oct4 overexpression, ^‡^*P*<0.05 *versus* HIF-2*α* or Oct4 silencing, ^§^*P*<0.05 *versus* HIF-2*α* and Oct4 co-overexpression, ^||^*P*<0.05 *versus* HIF-2*α* overexpression and Oct4 silencing (*n*=10 per group). (C) HIF-2*α* and Oct4 expression in cells determined by immunofluorescence with anti-Oct4 (green) and anti-HIF-2*α* (red) antibodies, respectively. Also shown are DAPI staining (nuclei; blue) and merged images. Bars=10 *μ*m. HIF-2*α* and Oct4 were mainly localized in the nucleus. HIF-2*α* or Oct4 overexpression markedly increased the staining intensity of HIF-2*α* and Oct4, while HIF-2*α* or Oct4 silencing markedly suppressed it. The increase was further improved in the cells co-overexpressing both HIF-2*α* and Oct4, and an inhibitory effect was observed when one transcription factor was overexpressed and the other was silenced. These data all indicate the physical co-binding of HIF-2*α* and Oct4. (**b**) Proliferation was evaluated by Ki67-positive cells under immunofluorescence microscopy; (**c**) cell death was evaluated via flow cytometry analysis of annexin V-stained cells; (**d**) HIF-2*α*, Oct4, bFGF, VEGF, Bcl2, survivin, and caspase-3 mRNA and protein levels were evaluated with qRT-PCR (A) and western blotting (B), respectively. *P<0.05 *versus* vehicle, ^†^*P*<0.05 *versus* HIF-2*α* or Oct4 overexpression, ^‡^*P*<0.05 *versus* HIF-2*α* or Oct4 silencing, ^§^*P*<0.05 *versus* HIF-2*α* and Oct4 co-overexpression, ^||^*P*<0.05 *versus* HIF-2*α* overexpression and Oct4 silencing (*n*=10 per group)

Figures 4aA and B show that Oct4 and HIF-2*α* mRNA and protein levels were significantly upregulated by Oct4 or HIF-2*α* overexpression and were downregulated by HIF-2*α* or Oct4 siRNA inhibition. Co-overexpressing HIF-2*α* and Oct4 further increased HIF-2*α* and Oct4 expression, but overexpressing one transcription factor while silencing the other only caused a corresponding increase in the expression of the overexpressed gene and decreased the expression of the silenced gene. These changes were confirmed by immunofluorescence (Figure 4aC).

Under hypoxic conditions, vselMSCs overexpressing HIF-2*α* or Oct4 were significantly more proliferative than ^WT^vselMSCs, and co-overexpressing HIF-2*α* and Oct4 further promoted cell proliferation. HIF-2*α* or Oct4 siRNAs led to a greater antiproliferative effect, and overexpressing one transcription factor while silencing the other produced the same effects (Figure 4b). The apoptotic ratios were lowest in HIF-2*α*^+^Oct4^+^ cells, second lowest in cells overexpressing HIF-2*α* or Oct4 alone, and highest in cells treated with HIF-2*α* or Oct4 siRNAs with or without Oct4/HIF-2*α* overexpression (Figure 4c), suggesting that HIF-2*α* and Oct4 cooperatively protects vselMSCs against the apoptotic response to hypoxic injury.

There was high expression of bFGF, VEGF, Bcl2, and survivin mRNA and protein in ^HIF-2*α*+^vselMSCs and ^Oct4+^vselMSCs as compared to ^WT^vselMSCs, and much more than in ^siHIF-2*α*^ vselMSCs and ^siOct4^ vselMSCs, respectively; however, measurements in HIF-2*α* or Oct4-deficient cells with or without Oct4/HIF-2*α* overexpression were similar and generally lower than in cells with unmodified Oct4 and HIF-2*α* expressions. Caspase 3 expression was lower in ^WT^vselMSCs than in ^siHIF-2*α*^ vselMSCs and ^siOct4^ vselMSCs, and was downregulated in HIF-2*α* or Oct4-overexpressing cells (Figures 4dA and B). These data all show that Oct4 and HIF-2*α* cooperatively share many anti-apoptotic transcriptional targets.

### Oct4 collaborates with HIF-2*α* to regulate vselMSCs pluripotency under hypoxia

**Figure 5:**
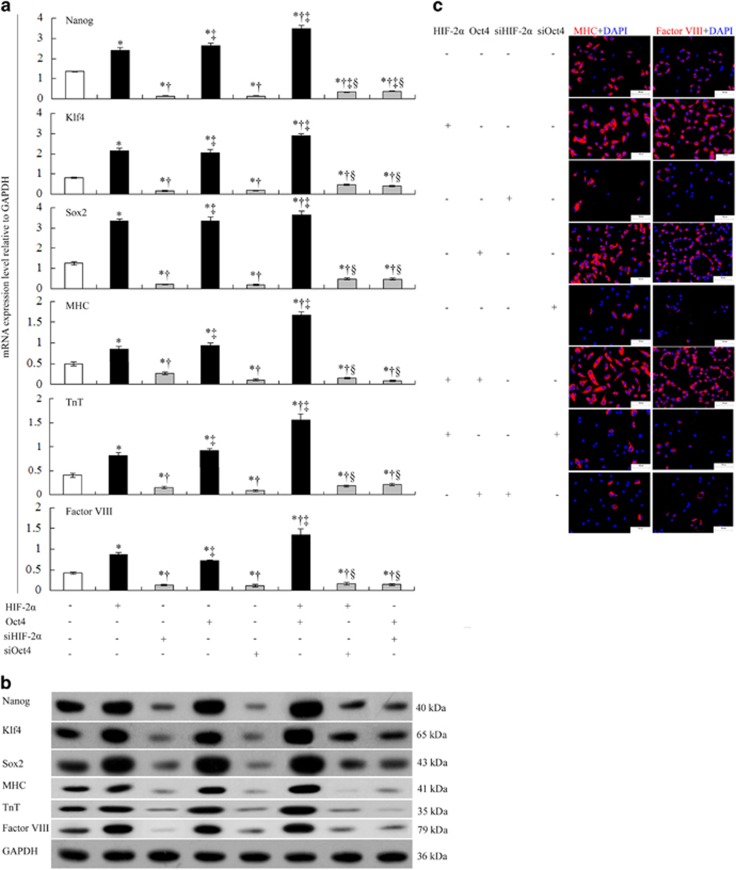
Effects of HIF-2α and Oct4 on induced differentiation in vselMSCs*.* Under hypoxic conditions, vselMSCs with enhanced, deficient, or WT levels of HIF-2*α* or Oct4 activity were examined for differences in (**a**) mRNA (qRT-PCR) and (**b**) protein levels (western blotting) of the pluripotency factors Nanog, Klf4, and Sox2, the cardiomyocyte markers MHC and troponin T (TnT), and the endothelial marker factor VIII. *P<0.05 *versus* vehicle, ^†^*P*<0.05 *versus* HIF-2*α* or Oct4 overexpression, ^‡^*P*<0.05 *versus* HIF-2*α* or Oct4 silencing, ^§^*P*<0.05 *versus* HIF-2*α* and Oct4 co-overexpression (*n*=10 per group). (**c**) Cell differentiation was induced by growth factor treatment. MHC and factor VIII expression was visualized in treated cells by immunofluorescence (bars=50 *μ*m). The nuclei were stained with DAPI (blue), and the cytoplasm of the myocardiocytes or blood endothelial cells was stained red with anti-MHC or anti-factor VIII, respectively

Compared with ^WT^vselMSCs, HIF-2*α* or Oct4 overexpression alone upregulated mRNA and protein expressions of Klf4, Nanog, and Sox2 in vselMSCs, and HIF-2*α* and Oct4 co-overexpression further improved this upregulation; siHIF-2*α* or siOct4 abolished the upregulation, and overexpressing one transcription factor while silencing the other elicited the same results (Figures 5a and b). Compared with those in ^WT^vselMSCs, the mRNA and protein expression levels of MHC, troponin T and factor VIII were highest in vselMSCs co-overexpressing HIF-2*α* and Oct4, followed by that in vselMSCs overexpressing either one transcription factor, and were significantly lower in HIF-2*α*- or Oct4-deficient cells combined with Oct4 or HIF-2*α* overexpression (Figures 5a and b). HIF-2*α* and Oct4 overexpression together showed the same change in the number of vselMSCs that expressed cardiomyocyte and/or vascular cell markers (Figure 5c).

### HIF-2*α* and Oct4 cooperate to promote myocardial repair induced by vselMSCs therapy

**Figure 6:**
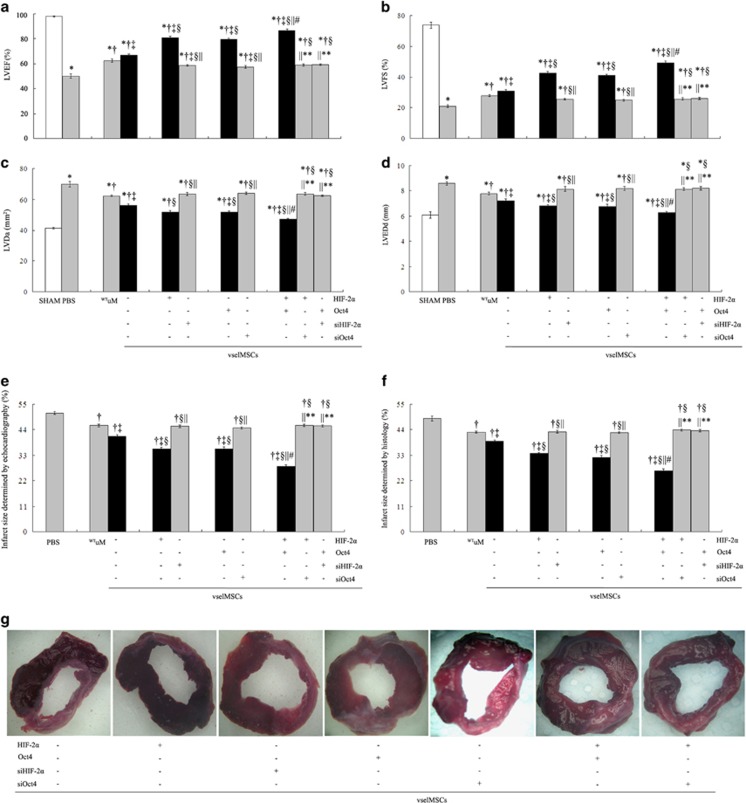
Collaboration of HIF-2*α* and Oct4 increases the functional and structural benefits of vselMSC transplantation in hearts with ischemic injury. MI was surgically induced in rats, and then saline (PBS), uMSCs, or vselMSCs with enhanced, deficient, or WT levels of HIF-2*α* or Oct4 activity were injected into the infarcted regions. Echocardiographic assessments of (**a**) left-ventricular (LV) ejection fraction (LVEF), (**b**) fractional shortening (LVFS), (**c**) diastolic area (LVDa), (**d**) diastolic diameter (LVEDd), and infarct size determined by echocardiography (**e**) and histology (**f**) were performed 30 days later. *P<0.05 *versus* SHAM, ^†^*P*<0.05 *versus* PBS, ^‡^*P*<0.05 *versus* ^WT^uM, ^§^*P*<0.05 *versus* vehicle vselMSCs, ^||^*P*<0.05 *versus* vselMSCs overexpressing HIF-2*α* or Oct4, ^#^*P*<0.05 *versus* vselMSCs with HIF-2*α* or Oct4 silencing, **P<0.05 *versus* HIF-2*α* and Oct4 co-overexpression (SHAM, *n*=10; PBS, *n*=12; ^WT^uM, *n*=12; ^WT^vselMSCs, *n*=13; ^HIF-2*α*+^vselMSCs, *n*=14; ^siHIF-2*α*+^vselMSCs, *n*=11; ^Oct4+^vselMSCs, *n*=14; ^siOct4+^vselMSCs, *n*=12; ^HIF-2+*α*Oct4+^vselMSCs, *n*=15; ^HIF-2+*α*siOct4+^vselMSCs, *n*=12; ^Oct4+siHIF-2*α*+^vselMSCs, *n*=13). (**c**) Cell differentiation was induced by growth factor treatment. MHC and factor VIII expression was visualized in treated cells by immunofluorescence (bars=50 *μ*m). (**g**) TTC-stained and cut into transverse sections to assess infarct size (percentage of the area of the entire LV). None of the infarcted myocardium was stained red by TTC; the pale region is the infarcted myocardium

Echocardiography revealed significant deterioration in the LV function and structural indices in all MI animals that had received PBS injection or cell transplantation in comparison with the SHAM group (Figures 6a–d). However, all functional and structural parameters were significantly better in animals treated with cells expressing unmodified levels of HIF-2*α* and Oct4 than in saline-treated animals (PBS group), in ^WT^vselMSC (^WT^vsel)-treated animals than in the ^WT^uMSC (^WT^uM)-treatment group, and in ^HIF-2*α*^vselMSC or ^Oct4^vselMSC-treated than in ^siHIF-2*α*^vselMSC or ^siOct4^vselMSC-treated animals. The greatest improvement was seen in the vselMSCs transfected with both HIF-2*α* and Oct4, and overexpressing one transcription factor while silencing the other caused obviously decreased effects (Figures 6a–g).

### Overexpression of HIF-2*α* and Oct4 enhance angiogenesis induced by vselMSCs transplantation

**Figure 7:**
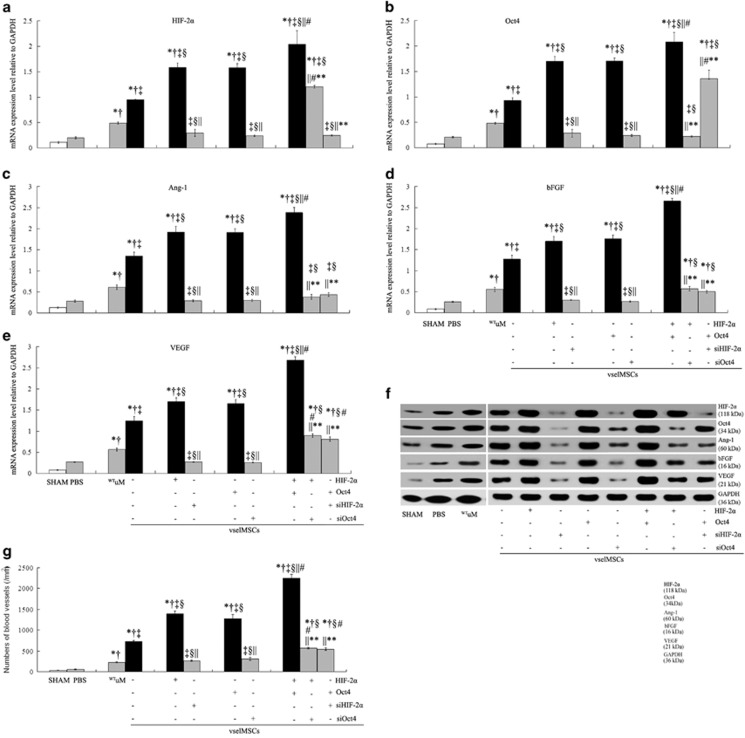

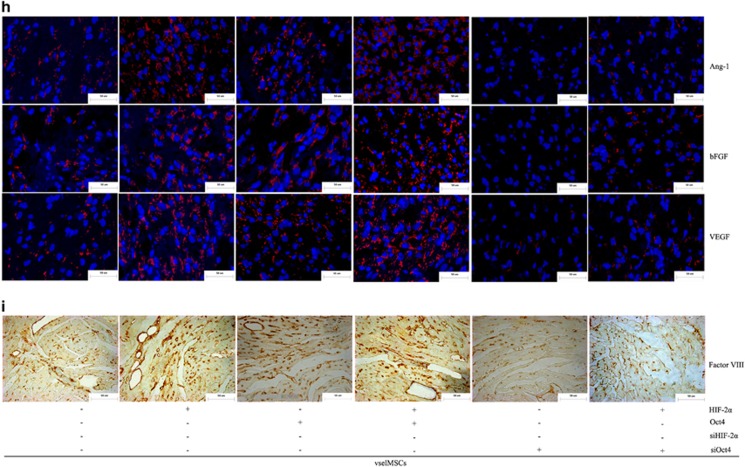
Identification of target genes coregulated by HIF-2α and Oct4 on angiogenesis of transplanted vselMSCs. mRNA (qRT-PCR) of HIF-2*α* (**a**) and Oct4 (**b**) and of the proangiogenic proteins angiopoietin 1 (Ang-1, **c**), bFGF (**d**), and VEGF (**e**) in sections from the SHAM rat hearts, and the peri-infarct regions of rats treated with saline (PBS), uMSC and with vselMSCs with enhanced, deficient, or WT levels of HIF-2*α* or Oct4 activity. (**f**) Representative western blots of HIF-2*α*, Oct4, Ang-1, bFGF, and VEGF levels in rat hearts 1 month post-operation. (**g**) show the quantitative analysis of vessel density by staining with factor VIII. *P<0.05 *versus* SHAM, ^†^*P*<0.05 *versus* PBS, ^‡^*P*<0.05 *versus* ^WT^uM, ^§^*P*<0.05 *versus* vehicle vselMSCs, ^||^*P*<0.05 *versus* vselMSCs overexpressing HIF-2*α* or Oct4, ^#^*P*<0.05 *versus* vselMSCs with HIF-2*α* or Oct4 silencing, **P<0.05 *versus* HIF-2*α* and Oct4 co-overexpression (SHAM, *n*=5; PBS, *n*=7; ^WT^uM, *n*=7; ^WT^vselMSCs, *n*=8; ^HIF-2*α*+^vselMSCs, *n*=9; ^siHIF-2*α*+^vselMSCs, *n*=6; ^Oct4+^vselMSCs, *n*=9; ^siOct4+^vselMSCs, *n*=7; ^HIF-2*α*+Oct4+^vselMSCs, *n*=10; ^HIF-2*α*+siOct4+^vselMSCs, *n*=7; ^Oct4+siHIF-2*α*+^vselMSCs, *n*=8). (**h**) Immunofluorescence of expression of the proangiogenic factors Ang-1, bFGF, and VEGF in peri-infarct regions via the corresponding antibodies (red), the nuclei were stained blue with DAPI (bars=50 *μ*m). Ang-1, bFGF and VEGF were mainly expressed by the blood vessels and cardiomyocytes in the vselMSCs-treated animals, especially in those receiving ^HIF-2*α*^vselMSCs or ^Oct4^vselMSCs transplantation, and more obviously in the animals that had received vselMSCs combined with HIF-2*α* and Oct4 transfection. (**i**) Evaluation of vascularity in the peri-infarct regions via immunostaining for factor VIII expression (brown); quantification was performed by counting positively stained vascular structures (bars=50 *μ*m)

Compared with SHAM and PBS injection, uMSCs transplantation resulted in increase of HIF-2*α* and Oct4 mRNA expression in the infarcted hearts, and vselMSCs therapy significantly increased their expressions. vselMSC transplantation combined with the transfection of either HIF-2*α* or Oct4 further increased mRNA expression of both HIF-2*α* and Oct4, and vselMSC transplantation combined with HIF-2*α* and Oct4 transfection led to the greatest increase (Figures 7a and b). Furthermore, the expression level of HIF-2*α* induced by transplantation of vselMSCs transfected with HIF-2*α* and siOct4 was lower than that of vselMSCs therapy combined with transfection of HIF-2*α* alone and *vice versa*.

Proangiogenic factors, Ang-1, bFGF, and VEGF in the group receiving vselMSCs alone were expressed at higher levels than in the ^WT^uMSC, PBS, and SHAM groups. HIF-2*α* or Oct4 overexpression further increased the mRNA expressions of those factors, and the HIF-2*α* and Oct4 combination caused the greatest increase, which were significantly reduced by HIF-2*α* or Oct4 deficiency, or siHIF-2*α* or siOct4 transfection combined with Oct4 or HIF-2*α* overexpression. Although Ang-1 and bFGF expression was not significantly different between HIF-2*α*- or Oct4-deficient cells and between cells transfected with siHIF-2*α* or siOct4 combined with Oct4 or HIF-2*α* overexpression, VEGF mRNA expression was significantly higher in the latter group (Figures 7c–e). Immunoblots showed the same trends of those factors (Figure 7f). These results were confirmed by immunofluorescence (Figure 7h). There was no significant difference between the SHAM group and PBS group. Collectively, these findings suggested that the initial upregulation of the expressions of proangiogenic cytokines is induced by vselMSCs transplantation, and overexpression of HIF-2*α* and Oct4 enhances this upregulation.

The numbers of blood vessels stained for anti-factor VIII antibody were greater in the rats that received vselMSC therapy than in the uMSC and the PBS groups, the greatest in the vselMSCs transfected with HIF-2*α* and Oct4, followed by that in the rats that had received vselMSCs transfected with HIF-2*α* or Oct4 alone, and were significantly reduced in the rats that had received vselMSCs combined with siHIF-2*α* or siOct4 transfection combined with or without Oct4 or HIF-2*α* overexpression (Figures 7g and i).

### Overexpression of HIF-2*α* and Oct4 protects vselMSCs against ischemia-induced injury

**Figure 8:**
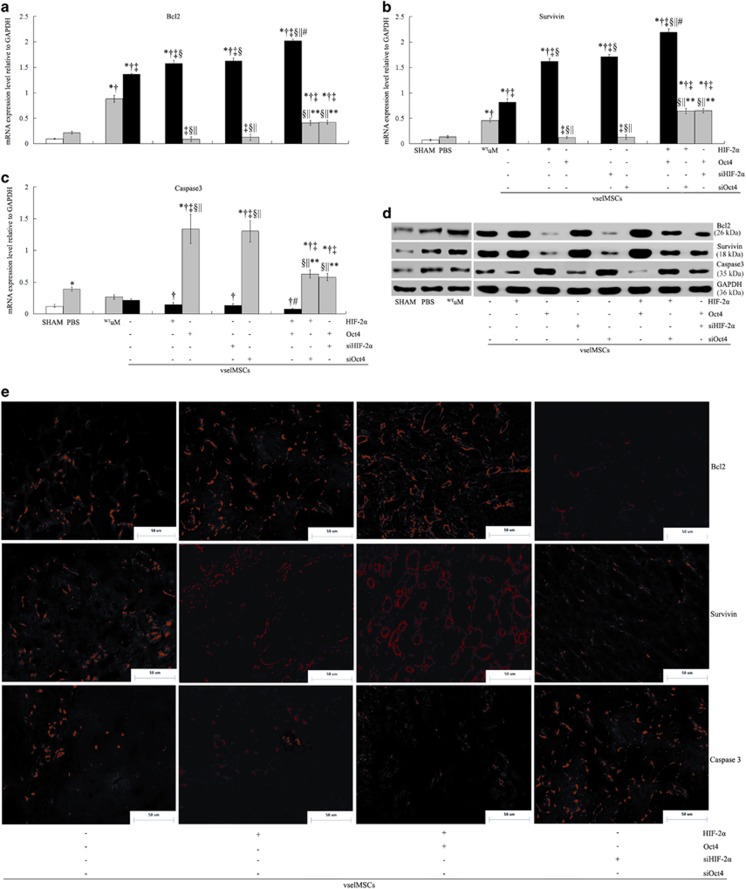
HIF-2α and Oct4 regulate the expression of anti-apoptotic genes*.* mRNA expression levels of the anti-apoptotic proteins Bcl-2 (**a**) and survivin (**b**) and the pro-apoptotic protein caspase 3 (**c**) evaluated in sections from the peri-infarct regions of rats treated with sham operation (SHAM), saline (PBS), with vselMSCs with enhanced, deficient, or WT levels of HIF-2*α* or Oct4 activity. *P<0.05 *versus* SHAM, ^†^*P*<0.05 *versus* PBS, ^‡^*P*<0.05 *versus* ^WT^uM, ^§^*P*<0.05 *versus* vehicle vselMSCs, ^||^*P*<0.05 *versus* vselMSCs overexpressing HIF-2*α* or Oct4, ^#^*P*<0.05 *versus* vselMSCs with HIF-2*α* or Oct4 silencing, **P<0.05 *versus* HIF-2*α* and Oct4 co-overexpression (SHAM, *n*=5; PBS, *n*=7; ^WT^uM, *n*=7; ^WT^vselMSCs, *n*=8; ^HIF-2*α*+^vselMSCs, *n*=9; ^siHIF-2*α*+^vselMSCs, *n*=6; ^Oct4+^vselMSCs, *n*=9; ^siOct4+^vselMSCs, *n*=7; ^HIF-2*α*+Oct4+^vselMSCs, *n*=10; ^HIF-2*α*+siOct4+^vselMSCs, *n*=7; ^Oct4+siHIF-2*α*+^vselMSCs, *n*=8). (**d**) Western blotting of Bcl-2, survivin, and caspase-3 expression levels. Protein expression correlated with mRNA expression. (**e**) Bcl2, survivin, and caspase 3 protein expression visualized in the peri-infarct regions from the ^WT^vsel, HIF-2α^+^, ^+^HIF-2α^+^Oct4^+^, and siHIF-2α^+^ via immunofluorescence staining with the corresponding antibodies (red) (bars=50 *μ*m)

Both anti-apoptotic (survivin and Bcl2) mRNA expression and protein expression were higher, and caspase 3 expression was lower, in ^WT^vselMSC- and ^WT^uMSC- treated hearts than in hearts from the PBS group, in ^WT^vselMSC-treated hearts than in ^WT^uMSC-treated hearts, and was improved in the ^HIF-2*α*+^vselMSC- and ^Oct4+^vselMSC-treated hearts; improvement was greatest in the ^HIF-2*α*+Oct4+^vselMSC-treated hearts (Figures 8a–d). Immunofluorescence showed that the numbers of the cells that expressed these anti-apoptotic proteins increased in response to HIF-2*α* and Oct4 overexpression, while caspase 3 expression declined (Figure 8e).

### HIF-2*α* and Oct4 overexpression enhanced the proliferation and engraftment of transplanted cells

**Figure 9:**
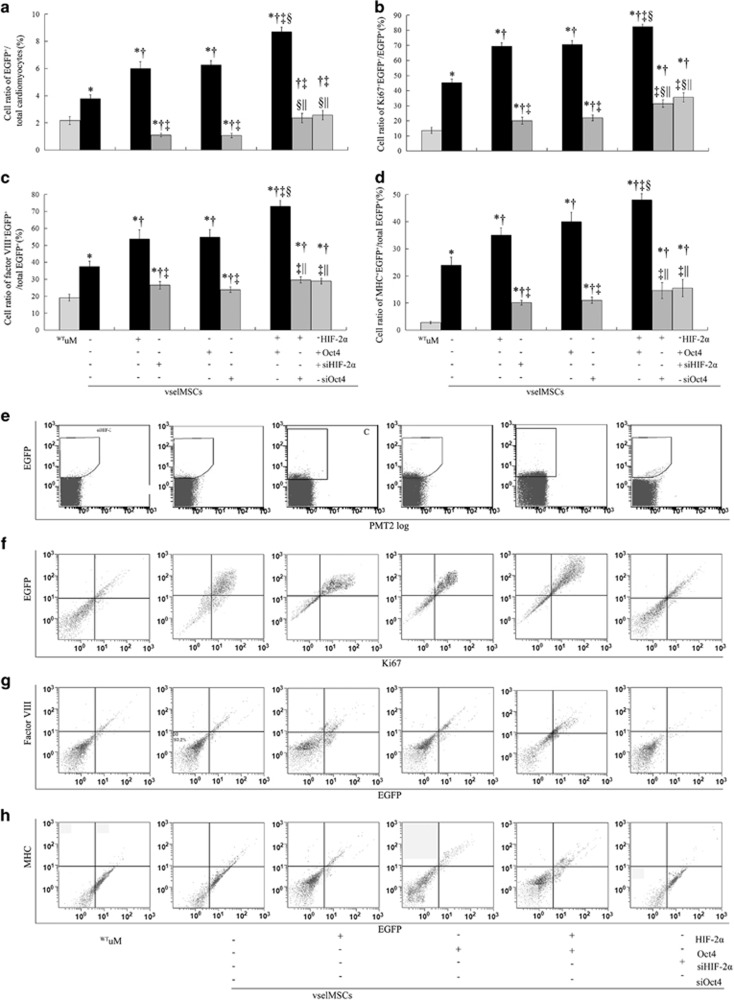

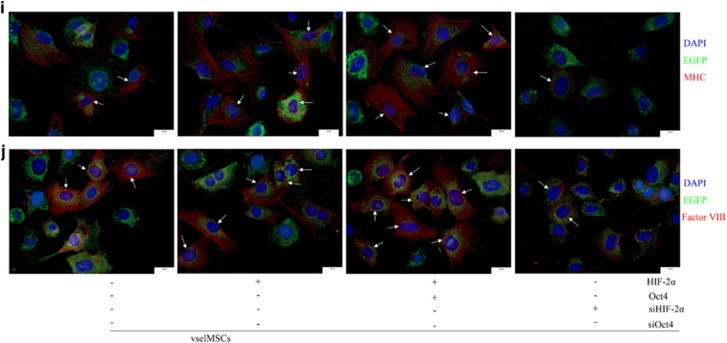
HIF-2α and Oct4 increase the proliferation and engraftment of transplanted vselMSCs. (**a**–**d**) Statistical analysis of the mean percentage of EGFP-positive cells (EGFP^+^) relative to the whole ventricular cell population (**a**), Ki67 and EGFP double-positive cells (Ki67^+^EGFP^+^) relative to the whole EGFP^+^ population (**b**), MHC and EGFP double-positive cells (MHC^+^EGFP^+^) relative to the whole EGFP^+^ population (**c**), and factor VIII and EGFP double-positive cells (factor VIII^+^EGFP^+^) relative to the whole EGFP^+^ population (**d**) as assessed by FACS. *P<0.05 *versus* ^WT^uM, ^†^*P*<0.05 *versus* vehicle vselMSCs, ^‡^*P*<0.05 *versus* HIF-2*α* or Oct4 overexpression, ^§^*P*<0.05 *versus* vselMSCs with HIF-2*α* or Oct4 silencing, ^||^*P*<0.05 *versus* HIF-2*α* and Oct4 co-overexpression (*n*=5 per group). (**e**–**h)** Representative phenotype of gated EGFP^+^ (**e**), Ki67^+^EGFP^+^ (**f**), MHC^+^EGFP^+^ (**g**), and factor VIII^+^EGFP^+^ cells (**h**) evaluated by FACS in ^WT^uM, vehicle vselMSCs, HIF-2*α*- or Oct4-overexpressing vselMSCs, and HIF-2*α*- or Oct4-silenced vselMSCs. (**i** and **j**) Immunofluorescence staining showing that transplanted cells expressed MHC (**i**) and factor VIII (**j**). The transplanted cells were pre-labeled with EGFP (green); the nuclei were stained with DAPI (blue), and the cytoplasm of the myocardiocytes or blood endothelial cells was stained red with anti-MHC or anti-factor VIII, respectively. Engrafted EGFP-pre-labeled cells expressing MHC or factor VIII were the most numerous in the ^HIF-2*α*+siOct4+^vselMSCs, followed by that in cells overexpressing HIF-2*α* or Oct4, and were lowest in HIF-2*α*- or Oct4-silenced vselMSCs (arrows)

EGFP-expressing cells were significantly more common in ^WT^vselMSC-treated hearts than in ^WT^uMSC-treated hearts, and were more common in ^HIF-2*α*+Oct4+^vselMSC-treated hearts than in the hearts with HIF-2*α* or Oct4 overexpression alone, while HIF-2*α* or Oct4 deficiency significantly decreased engraftment as compared with that in ^WT^vselMSCs (Figures 9a and e). More cells coexpressed EGFP and the proliferation marker Ki67 in ^WT^vselMSCs than in ^WT^uMSCs, which was significantly elevated by HIF-2*α* or Oct4 overexpression and was significantly reduced by HIF-2*α* or Oct4 deficiency (Figures 9b and f). This cooperation was reduced by siHIF-2*α* or siOct4 transfection combined with or without Oct4 or HIF-2*α* overexpression.

Expression of MHC and VIII was higher in ^WT^vselMSC-treated hearts than in ^WT^uMSC-treated hearts, in ^HIF-2*α*+^vselMSC- and ^Oct4+^vselMSC-treated hearts than in ^WT^vselMSC-treated hearts, and was further improved in hearts treated with vselMSCs co-overexpressing HIF-2*α* and Oct4 (Figures 9c, d and g–j).

## Discussion

This is the first study to demonstrate that vselMSCs isolated and purified from the coronary arterial blood MNCs and MSCs have greater survival and enhanced functions under hypoxic conditions compared with uMSCs. In addition, HIF-2*α* and Oct4 signaling improves cardiac function and remodeling induced by vselMSCs therapy after MI, and this myocardial repair can be significantly altered by HIF-2*α* or Oct4 overexpression and HIF-2*α* or Oct4 deficiency. We also observed the collaborative induction of angiogenesis, differentiation, and anti-apoptosis in vselMSCs by HIF-2*α* and Oct4. Taken together, the findings of this study suggested that collaboration between HIF-2*α* and Oct4 promotes survival and myocardial repair by human vselMSCs in MI.

Although human VSELs are enriched for CD133^+^Lin^−^CD45^−^ cells, and express stem cell markers such as Oct4, Nanog, and stage-specific embryonic antigen-4,^17^ the precise combination of markers can be affected by the isolation method and the presence of pathological conditions.^18, 19^ Some cells with morphological similarities to VSELs, such as those purified from umbilical cord blood of healthy patients with full-term pregnancies,^20^ fail to respond to ESC culture conditions. In this report, we used micromagnetic bead selection, multiparameter flow cytometry, limited-dilution culture and ESC culture expansion to obtain a VSELs subpopulation isolated from blood of the affected coronary artery in patients with MI. The isolated cells expressed unique molecular characteristics of mesenchymal, ESC and adult stem/progenitor cell markers, but were negative for hematopoietic (CD34 and Lineage) and monocyte-macrophage (CD45) marker expression. These cells have the VSELs' morphology as well as ESCs' pluripotency, which can be induced into three germ layers. Therefore, we called this population very small embryonic-like mesenchymal stem cells (vselMSCs). Moreover, the number of these vselMSCs in the infarct related artery was much higher than in those from the peripheral vein. This is the first report of a VSELs content difference in circulating blood MNCs from the culprit coronary artery compared with the peripheral vein.

Although myocardial transfection of HIF-1*α* and co-transplantation of mesenchymal stem cells could decrease the infarct size, and prevent post-infarction remodeling of the heart,^21^ but the role of HIF-2*α* in cell-autonomous VSEL maintenance remains unknown. The present study was the first to observe that HIF-2*α* expression in vselMSCs isolated and purified from a hypoxic environment was distinct from the uMSCs cultured in a normoxic environment, where only HIF-1*α* is expressed. By contrast, HIF-1*α* and HIF-2*α* are simultaneously highly expressed in vselMSCs, and we identified Oct4 as a novel collaborative interacting partner protein of HIF-2*α* in vselMSCs.

Recent studies suggest that HIF-2*α* positively regulates the transcriptional activity of Oct4 and enhances the physiological roles of Oct4.^22^ We demonstrated that HIF-2*α* genome occupancy in vselMSCs was similar to that in hESCs, and HIF-2*α* motifs were found to be enriched adjacent to the Oct4 motifs in vselMSCs. HIF-2*α* and Oct4 both accumulate in and around the nucleus and the cytoplasm under hypoxia-like conditions, and were upregulated in vselMSCs transfected with HIF-2*α* or Oct4, which reflected cell number increase and proliferation, and correlated negatively with the apoptotic rate under hypoxic culture or in ischemic hearts. Co-overexpressing HIF-2*α* and Oct4 together further enhanced the expression of both transcription factors and cell proliferation in both cultured vselMSCs *in vitro* and transplanted vselMSCs *in vivo*; silencing one transcription factor while overexpressing the other greatly weakened these effects. This result could aid in clarifying the synergistic effects of HIF-2*α* and Oct4 in improving cell growth during hypoxic or ischemic conditions.

Next, this coordination between HIF-2*α* and Oct4 was reflected in their regulation of vselMSC pluripotency and therapeutic potential. Overexpressing HIF-2*α* or Oct4 significantly increased the expression of the multipotency markers Klf4, Nanog, and Sox2; the cardiomyocyte markers MHC and troponin T; and the blood vascular endothelial cell marker factor VIII in the vselMSCs, and co-transfection with HIF-2*α* and Oct4 further enhanced this increase. Especially after transplantation into the ischemic hearts, HIF-2*α* and Oct4 expression were significantly higher in the infarcted hearts receiving vselMSCs therapy than in those receiving PBS injection or sham operation. These differences were consistently associated with improvements in cardiac function and left-ventricular structural remodeling of hearts treated with vselMSCs after MI. These effects were generally magnified by HIF-2*α* and Oct4 overexpression induced by HIF-2*α* or Oct4 transfection alone, and were further improved by the transfection of HIF-2*α* and Oct4 together. HIF-2*α* or Oct4 deficiency abolished these effects and significantly reduced the magnification induced by Oct4 or HIF-2*α* overexpression, respectively. Thus, the benefit of vselMSCs transplantation appears to be inextricably linked with the extent of HIF-2*α* and Oct4 coactivation, which is similar to the observation of Covello *et al.*^9^ that Oct4, as a HIF-2*α*-specific target gene, can regulate embryonic primordial germ cell function, which in turn contributes to HIF-2*α*'s tumor promoting activity.

On the other hand, the cooperative relationship between HIF-2*α* and Oct4 upregulated their target genes, including that for the proangiogenic factors Ang-1, bFGF, and VEGF; and the anti-apoptotic (survivin and Bcl2) and pro-apoptotic (caspase-3) proteins. The upregulation of these cytokines induced by HIF-2*α* and Oct4 overexpression was associated with significant promotion of the ratio of vselMSCs cultured *in vitro* differentiating into blood vascular endothelial cells (vasculogenesis) or vselMSCs engrafted into the infarcted hearts developing new blood vessels (angiogenesis). These data demonstrate that HIF-2*α* and Oct4 jointly regulate the expression of endogenous vascular permeabilizing factors, which are the target genes of HIF-2*α*-mediated angiogenesis under ischemic conditions.^23, 24^ The interaction between HIF-2*α* and Oct4 in regulating the anti-apoptotic and pro-apoptotic proteins was consistent with altered survival and apoptosis of vselMSCs after HIF-2*α* or Oct4 co-overexpression, overexpression of either transcription factor alone, or the silencing of both. Our results are similar to that of the study of Donskow-Łysoniewska *et al.*^25^ in that cell proliferation and apoptosis were dependent on a low Bax/Bcl-2 ratio, and upregulation of survivin, with inhibition of active caspase-3.

In conclusion, our findings underscore the likelihood that collaboration of HIF-2*α* and Oct4 must be considered not only as a part of the native physiological mechanisms that enhance stem cell survival and functions, but also act as potential synergists with vselMSCs therapy. In this manner, vselMSCs overexpressing HIF-2*α* and Oct4 may serve as an optimal donor for myocardial repair post-MI, and this area of physiology represents a potential therapeutic target for the future treatment of ischemic diseases.

## Materials and Methods

An expanded Methods section containing details regarding the patient population, fluorescence-activated cell sorting analysis, vselMSCs isolation, expansion, purification, and *in vitro* directed differentiation, immunocytofluorescence, microarray analysis, HIF-2*α* and Oct4 transfection, hypoxic treatment, cell viability and apoptosis analysis, green fluorescent protein (GFP) labeling, MI model and treatment, echocardiography, measurement of body weight ratio and infarct size, histology, immunofluorescence, real-time quantitative reverse transcription-PCR (qRT-PCR), immunoblotting, and statistics is available in the Online Data Supplement.

### Patient population

We studied ten 20- to 60-year-old patients with acute ST-segment elevation MI (STEMI) referred within 12 h after the symptomatic onset for primary percutaneous coronary intervention (PCI). To evaluate whether vselMSCs decline with age in the peripheral blood (PB) of the enrolled patients with AMI, 10 patients with STEMI aged >60–75 years were enrolled as controls. All patient-related procedures were performed with informed consent and in accordance with the guidelines of the Southern Medical University Committee on the Use of Human Subjects in Research.

### Fluorescence-activated cell sorting analysis of circulating blood mononuclear cells (MNCs)

Immediately after PCI, 10 ml circulating blood was collected from the peripheral vein and the culprit coronary artery, respectively. Fluorescence-activated cell sorting (FACS) analysis was performed to determine the lineage^−^CD45^−^CD133^+^ cell content in these MNCs.

### Isolation, expansion and purification of vselMSCs, and culture of unpurified MSCs (uMSCs) and ESCs

Supplementary Figure S1 shows the protocol of VSEL isolation and analysis. The vselMSCs were isolated and purified from the isolated MNCs as previously described.^26, 27^ As a control, uMSCs were cultured from the same patient's blood MNCs. The uMSCs were obtained from the above-mentioned MNCs via the adherent culture method. The hESC line H7 was purchased from SIDANSAI Biotechnology CO. (Shanghai, China, 0204-001) and used as the positive control.

### FACS of vselMSCs

The vselMSCs were incubated in phosphate-buffered saline with antibodies against CD34, CD44, CD71, CD147, SH2, SH3, stage-specific embryonic antigen-4 (SSEA-4), CD45, Lineage, and CD133.

### *In vitro* directed differentiation of vselMSCs

The vselMSCs underwent directed differentiation toward the ectoderm, endoderm, and mesoderm by growth factor supplementation and growth on defined matrices. Confluent vselMSC colonies were detached by incubation with 1 mg/ml collagenase (Invitrogen, Carlsbad, CA, USA) for 30–60 min, and replated onto low-attachment 6-well plates (Fisher, Chino, CA, USA) in embryoid body (EB) medium consisting of DMEM-F12 (Invitrogen) supplemented with 15% defined fetal bovine serum (FBS; HyClone, Pittsburgh, PA, USA), 5% knockout serum replacement (Invitrogen), 1 mM l-glutamine (Invitrogen), 2 mM 2-mercaptoethanol, 0.1 mM NEAA (Invitrogen), and 1 mM penicillin/streptomycin (HyClone). For neural-directed differentiation, day 5 vselMSCs were plated on fibronectin (20 *μ*g/ml)-coated dishes and cultured in DMEM/F12 supplemented with N2 and B27 (Invitrogen), 10 ng/ml bFGF, 1 ng/ml insulin-like growth factor (IGF), 1 ng/ml platelet-derived growth factor *α* polypeptide, and 10 ng/ml epidermal growth factor (all from PeproTech, Rocky Hill, NJ, USA) for an additional 5–7 days.^28^ For mesoderm differentiation, vselMSCs were cultured for 8 days in Stem Line II medium (Sigma-Aldrich, San Diego, CA, USA) supplemented with 1 × CD lipid concentrates, 2 mM GlutaMAX, 1 × insulin-transferrin-selenium, penicillin/streptomycin (100 units/100 mg/ml) (all from Invitrogen), 400 *μ*M monothioglycerol, and 50 mg/ml ascorbic acid (Sigma-Aldrich). The following growth factors were added: 10 ng/ml bone morphogenetic protein (BMP-4; R&D Systems, Minneapolis, MN, USA), 5 ng/ml bFGF (Invitrogen), and 20 ng/ml VEGF (R&D Systems). For ectoderm differentiation, vselMSCs were treated with 100 ng/ml activin (PeproTech) for 1 day and with 1% FBS and 100 ng/ml activin for the next 2 days in DMEM/F12.^14^

### Immunocytofluorescence

Images were collected on each cover slip under a light microscope. For immunocytofluorescence, cells were incubated with primary antibodies: *β*-tubulin III, glial fibrillary acidic protein (GFAP), troponin T, myosin heavy chain (MHC), factor VIII, alpha smooth muscle actin (*α*-SMA), human serum albumin, and alpha-fetoprotein (AFP).

### Microarray analysis

To analyze anti-apoptotic genetic similarity between vselMSCs and hESCs, these cells were produced over three sequential independent passages and hybridized to six Affymetrix HG-U133A chips. We compared the similarities of HIF-mediated anti-apoptotic genes between vselMSCs and hESCs using GEarray Expression Analysis Suite software containing 112 genes.

### HIF-2*α* and Oct4 transfection

Retroviral plasmid vectors, pMXs, expressing HIF-2*α* or Oct4, were transfected with the viral packaging genes gag-pol into vselMSCs with the Fugene HD reagent, as directed by the manufacturer's instructions. HIF-2*α* or Oct4 siRNAs and control siRNA duplexes were transfected together with pRL-TK plasmid vector (Promega, Madison, WI, USA) containing the *Renilla reniformis* luciferase gene into vselMSCs with LipofectAMINE 2000, as described previously.^29^ To determine whether HIF-2*α* and Oct4 could cooperatively alter vselMSCs survival and proliferation, experiments were performed with vselMSCs expressing unmodified (serving as the wild type, WT) levels of Oct4 and HIF-2*α*, those overexpressing HIF-2*α* or Oct4 cells (HIF-2*α*^+^, Oct4^+^), or coexpressing HIF-2*α* and Oct4 (HIF-2*α*^+^Oct4^+^), with HIF-2*α* or Oct4 silencing (siHIF-2*α*^+^ and siOct4^+^), or overexpressing one transcription factor while the other was silenced (HIF-2*α*^+^siOct4^+^ or siHIF-2*α*^+^Oct4^+^). HIF-2*α* or Oct4 overexpression and HIF-2*α* or Oct4 deficiency were induced by transfecting the cells with vectors encoding HIF-2*α* or Oct4, or HIF-2*α* and Oct4 siRNAs, respectively, WT cells were transfected with control vectors.

### Hypoxic treatment

Cells were removed and exposed to hypoxic (1%) oxygen levels in a water-jacketed CO_2_ incubator. The hypoxic condition was maintained throughout the performance of all subsequent analyses.

### Analysis of cell proliferation and apoptosis

Cell proliferation was assessed by fluorescence staining for the proliferation marker Ki67 using FACS. Apoptotic cell death under normoxic and hypoxic conditions was evaluated through annexin V (Roche Diagnostic, Indianapolis, IN, USA) and propidium iodide (PI).

### GFP labeling

Twenty-four hours after transfection with the HIF-2*α* or Oct4, siHIF-2*α*/siOct4, or control siRNA vectors, cells were co-transfected with a lentiviral vector containing enhanced GFP cDNA, as described previously.^30^

### MI model and treatment

Myocardial infarction was induced in male Sprague Dawley rats (200–250 g), obtained from the Shanghai Animal Administration Center, by ligating the left anterior descending coronary artery. The animals were then randomized to receive saline injection or cell therapy. To determine the effects of HIF-2*α* and Oct4 collaboration in stimulating myocardial repair, we performed an animal study to transplant vselMSCs overexpressing HIF-2*α* or Oct4, and those with HIF-2*α* or Oct4 siRNA transfection into the infarcted hearts. 191 out of the 270 experimental animals survived the MI operation. 163 animals with an ejection fraction (EF) <70% and fractional shortening (FS) <35% received PBS or cell injection. 25 rats died of malignant arrhythmia, acute left-ventricular failure and ventricular perforation post injection and 138 animals survived to the scheduled study end (SHAM, *n*=10; PBS, *n*=12; ^WT^uM, *n*=12; ^WT^vselMSCs, *n*=13; ^HIF-2*α*+^vselMSCs, *n*=14; ^siHIF-2*α*+^vselMSCs, *n*=11; ^Oct4+^vselMSCs, *n*=14; ^siOct4+^vselMSCs, *n*=12; ^HIF-2*α*+Oct4+^vselMSCs, *n*=15; ^HIF-2*α*+siOct4+^vselMSCs, *n*=12; ^Oct4+siHIF-2*α*+^vselMSCs, *n*=13, respectively). There was no significant difference in mortality among the seven groups at the endpoint (*P*>0.5). No malignant arrhythmia was found on ECG recordings on scheduled study end and no tumor formation was observed at autopsy.

### Echocardiography

Thirty days later, cardiac functions were evaluated by echocardiographic assessments of LVEF, LVFS, LV diastolic area (LVDa), and diastolic diameter (LVEDd), and the structural benefits of therapy were evaluated by measuring the LV infarct size as determined by echocardiography.

### Histology and immunofluorescence

The left ventricles of the remaining rats were weighed to calculate the ratio of left-ventricular weight to body weight. The size of the infarct was obtained by calculating the percentage of the infarcted area against the whole LV area using a digital imaging program (Scion Image 4.03, Bethesda, MD, USA). The tissues from the autopsy specimens were embedded in paraffin or frozen for cryostat sectioning and were then stained by hematoxylin and eosin or used in immunofluorescence assays. For immunocytofluorescence, cells were fixed with fresh 4% paraformaldehyde in PBS.

### QRT-PCR and immunoblotting

The cells and the autopsied tissues were collected and pulverized to extract RNA or protein for qRT-PCR and immunoblotting. Table 1 lists the sequences of the primers and probes used to analyze the expression of the human and rat genes.

### Statistical analysis

The results are expressed as the mean±S.E.M. and were tested for significance using analysis of variance for multiple comparisons. Chi-square analysis was used to compare survival rates between groups. A *P*-value of <0.05 was considered statistically significant.

### Online data supplementary figures

Supplementary Figure S1 in the online-only Data Supplement presents the experimental flow of the vselMSC development, and analysis of cooperation between HIF-2*α* and Oct4 in regulating vselMSC pluripotency, survival, proliferation, and therapeutic potential.

## Figures and Tables

**Table 1:** The sequences of primers and probes for real-time RT-PCR

**Gene name**	**Sequence 5′-3′**	**Size (bp)**	**Accession No.**
hOct 4	TCTATTTGGGAAGGTATTCAGC ATTGTTGTCAGCTTCCTCCA	124	NM_002701.4
rOct 4	CCACTTCACCACACTCTACT GTCACCGCATGTTAGAAGAC	127	AC_000076
hNanog	CAGCTACAAACAGGTGAAGAC TGGTGGTAGGAAGAGTAAAGG	147	NM_024865.2
hKlf4	ATTACCAAGAGCTCATGCCA CCTTGAGATGGGAACTCTTTG	152	NM_004235.3
hSox2	GCCGAGTGGAAACTTTTGTCG GGCAGCGTGTACTTATCCTTCT	155	NM_003106
hHIF-1	GAACGTCGAAAAGAAAAGTCTCG CCTTATCAAGATGCGAACTCACA	124	NM_001530
hHIF-2	GGGCCAGGTGAAAGTCTACA TGCTGGATTGGTTCACACAT	105	NM_001430
rHIF-2	CCCCAGGGGATGCTATTATT GGCGAAGAGCTTCTCGATTA	298	AJ277828
hAng-1	AATGGACTGGGAAGGGAACC GCATCAAACCACCATCCTCCT	222	NM_001146.3
rAng-1	AACAGGAGGTTGGTGGTTTGATG GAACATCCCCAGATTTATTTCAGGT	217	NM_053546.1
hbFGF	CTCTGGGTAGGTGAGTTGTTGTGAC TGTGAACTCTTATGGGTCCTCTGA	213	NM_002006.4
rbFGF	CCATCAAGGGAGTGTGTGCG CCCAGTTCGTTTCAGTGCCA	176	NM_019305.2
hVEGF	GTGCCCACTGAGGAGTCCAACA TTAGACAGCAGCGGGCACCA	238	NM_001025366.2
rVEGF	CCGACAGGGAAGACAATGGGA GGGATGGGTTTGTCGTGTTTCT	149	NM_001110333.2
hSurvivin	AGCCCTTTCTCAAGGACCAC CCGCAGTTTCCTCAAATTCTT	236	NM_001012270.1
rSurvivin	CACTGCCCTACCGAGAATG TCTTCCACCTGCTTCTTGACT	152	NM_022274.1
hBcl2	CTGGAGAGTGCTGAAGATTGATGG TGATTCTGGTGTTTCCCCCTT	232	NM_000633.2
rBcl2	GGTGGAGGAACTCTTCAGGGAT GATGCCGGTTCAGGTACTCAGT	155	NM_016993.1
hCaspase3	AGCACTGGAATGACATCTCGGT GCTCAGAAGCACACAAACAAAACT	189	NM_004346.3
rCaspase3	TACCGATGTCGATGCAGCTAAC TTTTCAGGTCCACAGGTCCG	215	NM_012922.2
hGADPH	CTCATTTCCTGGTATGACAACGA CTTCCTCTTGTGCTCTTGCT	121	NM_002046.3
rGAPDH	CCGAGGGCCCACTAAAGG TGCTGTTGAAGTCACAGGAGACA	67	NM_017008

Abbreviations: Ang-1, angiopoietin 1; Bcl-2, B-cell lymphoma 2; bFGF, basic fibroblast growth factor; bp, base pair; GAPDH, glyceraldehyde-3-phosphate dehydrogenase; h, human; HIF-1, hypoxia-inducible factor 1; HIF-2, hypoxia-inducible factor 2; Klf4, Krüppel-like factor; Oct4, octamer-binding transcription factor 4; r, rat; VEGF, vascular endothelial growth factor.

Primers for real-time RT-PCR.

## References

[bib1] Perin EC, Borow KM, Silva GV, DeMaria AN, Marroquin OC, Huang PP et al. A phase II dose-escalation study of allogeneic mesenchymal precursor cells in patients with ischemic or nonischemic heart failure. Circ Res 2015; 117: 576–584. 26148930 10.1161/CIRCRESAHA.115.306332

[bib2] Dixit P, Katare R. Challenges in identifying the best source of stem cells for cardiac regeneration therapy. Stem Cell Res Ther 2015; 6: 26. 25886612 10.1186/s13287-015-0010-8 PMC4357059

[bib3] Williams AR, Hare JM. Mesenchymal stem cells: biology, pathophysiology, translational findings, and therapeutic implications for cardiac disease. Circ Res 2011; 109: 923–940. 21960725 10.1161/CIRCRESAHA.111.243147 PMC3604746

[bib4] Tran N, Li Y, Maskali F, Antunes L, Maureira P, Laurens MH et al. Short-term heart retention and distribution of intramyocardial delivered mesenchymal cells within necrotic or intact myocardium. Cell Transplant 2006; 15: 351–358. 16898229 10.3727/000000006783981918

[bib5] Chen YB, Lan YW, Chen LG, Huang TT, Choo KB, Cheng WT et al. Mesenchymal stem cell-based HSP70 promoter-driven VEGFA induction by resveratrol alleviates elastase-induced emphysema in a mouse model. Cell Stress Chaperones 2015; 20: 979–989. 26243699 10.1007/s12192-015-0627-7 PMC4595438

[bib6] Mingliang R, Bo Z, Zhengguo W. Stem cells for cardiac repair: status, mechanisms, and new strategies. Stem Cells Int 2011; 2011: 310928. 21776280 10.4061/2011/310928 PMC3137967

[bib7] Das H, George JC, Joseph M, Das M, Abdulhameed N, Blitz A et al. Stem cell therapy with overexpressed VEGF and PDGF genes improves cardiac function in a rat infarct model. PLoS One 2009; 4: e7325. 19809493 10.1371/journal.pone.0007325 PMC2752797

[bib8] Forristal CE, Wright KL, Hanley NA, Oreffo RO, Houghton FD. Hypoxia inducible factors regulate pluripotency and proliferation in human embryonic stem cells cultured at reduced oxygen tensions. Reproduction 2010; 139: 85–97. 19755485 10.1530/REP-09-0300 PMC2791494

[bib9] Covello KL, Kehler J, Yu H, Gordan JD, Arsham AM, Hu CJ et al. HIF-2 regulates Oct-4: effects of hypoxia on stem cell function, embryonic development, and tumor growth. Genes Dev 2006; 20: 557–570. 16510872 10.1101/gad.1399906 PMC1410808

[bib10] Iso Y, Rao KS, Poole CN, Zaman AK, Curril I, Sobel BE et al. Priming with ligands secreted by human stromal progenitor cells promotes grafts of cardiac stem/progenitor cells after myocardial infarction. Stem Cells 2014; 32: 674–683. 24022988 10.1002/stem.1546 PMC3966427

[bib11] Zhang S, Ge J, Sun A, Xu D, Qian J, Lin J et al. Comparison of various kinds of bone marrow stem cells for the repair of infarcted myocardium: single clonally purified non-hematopoietic mesenchymal stem cells serve as a superior source. J Cell Biochem 2006; 99: 1132–1147. 16795039 10.1002/jcb.20949

[bib12] Hnatiuk AP, Ong SG, Olea FD, Locatelli P, Riegler J, Lee WH et al. Allogeneic mesenchymal stromal cells overexpressing mutant human hypoxia-inducible factor 1-*α* (HIF1-*α* in an ovine model of acute myocardial infarction. J Am Heart Assoc 2016; 5: pii: e003714. 10.1161/JAHA.116.003714 PMC5015403 27385426

[bib13] Abdel-Latif A, Zuba-Surma EK, Ziada KM, Kucia M, Cohen DA, Kaplan AM et al. Evidence of mobilization of pluripotent stem cells into peripheral blood of patients with myocardial ischemia. Exp Hematol 2010; 38: 1131–1142. 20800644 10.1016/j.exphem.2010.08.003 PMC2992878

[bib14] Alva JA, Lee GE, Escobar EE, Pyle AD. Phosphatase and tensin homolog regulates the pluripotent state and lineage fate choice in human embryonic stem cells. Stem Cells 2011; 29: 1952–1962. 21948699 10.1002/stem.748 PMC3898662

[bib15] Wojakowski W, Kucia M, Liu R, Zuba-Surma E, Jadczyk T, Bachowski R et al. Circulating very small embryonic-like stem cells in cardiovascular disease. J Cardiovasc Transl Res 2011; 4: 138–144. 21165781 10.1007/s12265-010-9254-y PMC3047714

[bib16] Tseng TC, Hsieh FY, Dai NT, Hsu SH. Substrate-mediated reprogramming of human fibroblasts into neural crest stem-like cells and their applications in neural repair. Biomaterials 2016; 102: 148–161. 27341268 10.1016/j.biomaterials.2016.06.020

[bib17] Kurkure P, Prasad M, Dhamankar V, Bakshi G. Very small embryonic-like stem cells (VSELs) detected in azoospermic testicular biopsies of adult survivors of childhood cancer. Reprod Biol Endocrinol 2015; 13: 122. 26553338 10.1186/s12958-015-0121-1 PMC4640406

[bib18] Ratajczak MZ, Mierzejewska K, Ratajczak J, Kucia M. CD133 expression strongly correlates with the phenotype of very small embryonic-/epiblast-like stem cells. Adv Exp Med Biol 2013; 777: 125–141. 23161080 10.1007/978-1-4614-5894-4_9 PMC5565199

[bib19] Havens AM, Shiozawa Y, Jung Y, Sun H, Wang J, McGee S et al. Human very small embryonic-like cells generate skeletal structures, *in vivo*. Stem Cells Dev 2013; 22: 622–630. 23020187 10.1089/scd.2012.0327 PMC3564465

[bib20] Marlicz W, Zuba-Surma E, Kucia M, Blogowski W, Starzynska T, Ratajczak MZ. Various types of stem cells, including a population of very small embryonic-like stem cells, are mobilized into peripheral blood in patients with Crohn's disease. Inflamm Bowel Dis 2012; 18: 1711–1722. 22238186 10.1002/ibd.22875

[bib21] Huang B, Qian J, Ma J, Huang Z, Shen Y, Chen X et al. Myocardial transfection of hypoxia-inducible factor-1*α* and co-transplantation of mesenchymal stem cells enhance cardiac repair in rats with experimentalmyocardial infarction. Stem Cell Res Ther 2014; 5: 22. 24507665 10.1186/scrt410 PMC4055118

[bib22] Danova-Alt R, Heider A, Egger D, Cross M, Alt R. Very small embryonic-like stem cells purified from umbilical cord blood lack stem cell characteristics. PLoS One 2012; 7: e34899. 22509366 10.1371/journal.pone.0034899 PMC3318011

[bib23] Lim SY, Hsiao ST, Lokmic Z, Sivakumaran P, Dusting GJ, Dilley RJ. Ischemic preconditioning promotes intrinsic vascularization and enhances survival of implanted cells in an in *vivo* tissue engineering model. Tissue Eng Part A 2012; 8: 2210–2219. 10.1089/ten.tea.2011.0719 PMC3482871 22651554

[bib24] Woik N, Kroll J. Regulation of lung development and regeneration by the vascular system. Cell Mol Life Sci 2015; 72: 2709–2718. 25894695 10.1007/s00018-015-1907-1 PMC11113134

[bib25] Donskow-Łysoniewska K, Brodaczewska K, Doligalska M. Heligmosomoides polygyrus antigens inhibit the intrinsic pathway of apoptosis by overexpression of survivin and Bcl-2 protein in CD4 T cells. Prion 2013; 7: 319–327. 23787700 10.4161/pri.25008 PMC3904318

[bib26] Shin DM, Suszynska M, Mierzejewska K, Ratajczak J, Ratajczak MZ. Very small embryonic-like stem-cell optimization of isolation protocols: an update of molecular signatures and a review of current *in vivo* applications. Exp Mol Med 2013; 45: e56. 24232255 10.1038/emm.2013.117 PMC3849570

[bib27] Parte S, Bhartiya D, Patel H, Daithankar V, Chauhan A, Zaveri K et al. Dynamics associated with spontaneous differentiation of ovarian stem cells *in vitro*. J Ovarian Res 2014; 7: 25. 24568237 10.1186/1757-2215-7-25 PMC4234975

[bib28] Carpenter MK, Inokuma MS, Denham J, Mujtaba T, Chiu CP, Rao MS. Enrichment of neurons and neural precursors from human embryonic stem cells. Exp Neurol 2001; 172: 383–397. 11716562 10.1006/exnr.2001.7832

[bib29] Bhartiya D, Shaikh A, Nagvenkar P, Kasiviswanathan S, Pethe P, Pawani H et al. Very small embryonic-like stem cells with maximum regenerative potential get discarded during cord blood banking and bone marrow processing for autologous stem cell therapy. Stem Cells Dev 2012; 21: 1–6. 21780911 10.1089/scd.2011.0311

[bib30] Zhang S, Ge J, Zhao L, Qian J, Huang Z, Shen L et al. Host vascular niche contributes to myocardial repair induced by intracoronary bone marrow stem cells infusion in infarcted swine hearts. Stem Cells 2007; 25: 1195–1203. 17272498 10.1634/stemcells.2006-0605

