# Supplementary figures and images for: HIF-2α and Oct4 have synergistic effects on survival and myocardial repair of very small embryonic-like mesenchymal stem cells in infarcted hearts
Source: Cell Death Dis. 2017 Jan 12;8(1):e2548–. doi: 10.1038/cddis.2016.480 (PMC5386383; doi:10.1038/cddis.2016.480)

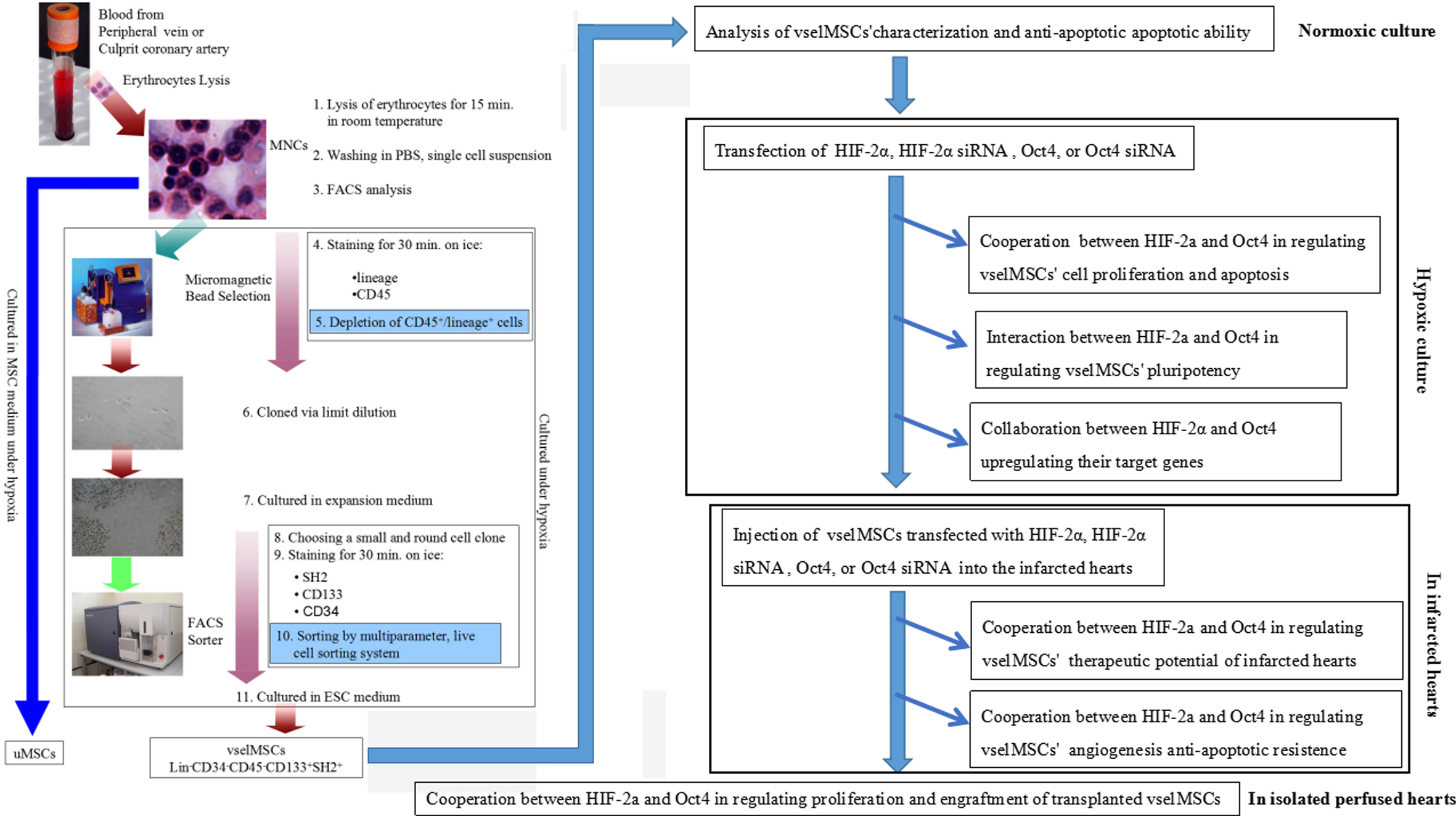

Supplement: Supplementary Figure S1 [file cddis2016480x1.tif]
