# Supplementary material for: HIF-2α and Oct4 have synergistic effects on survival and myocardial repair of very small embryonic-like mesenchymal stem cells in infarcted hearts
Source: Cell Death Dis. 2017 Jan 12;8(1):e2548–. doi: 10.1038/cddis.2016.480 (PMC5386383; doi:10.1038/cddis.2016.480)
Supplement: Supplementary Information [file cddis2016480x2.doc]

**Materials and methods**

**Patient population**

We studied 10 patients aged 20–60 years and with acute ST-segment elevation MI (STEMI) referred within 12 h after symptom onset for primary percutaneous coronary intervention (PCI). To evaluate whether vselMSCs decline with age in the peripheral blood (PB) of the enrolled patients with AMI, 10 patients aged >60–75 years and with STEMI were enrolled as controls. All patient-related procedures were performed with informed consent and in accordance with the guidelines of the Southern Medical University Committee on the Use of Human Subjects in Research that conform to the Declaration of Helsinki.

**Fluorescence-activated cell sorting (FACS) analysis of circulating VSELs** Immediately after PCI, 10 ml circulating blood was collected from the peripheral vein and the affected coronary artery each. MNCs were isolated from the samples using erythrocyte lysis buffer (BD Pharmingen, San Jose, CA, USA). To analyze the VSEL content in the circulating blood MNCs from the peripheral vein and the affected coronary artery, FACS analysis was performed to determine their lineage, i.e., CD45^-^CD133^+^ cell content ^[1]^. MNCs were washed twice with FACS buffer (phosphate-buffered saline [PBS] containing 0.1% BSA, 0.01% sodium azide, and 20 μg/ml aprotinin), and cell surface markers were evaluated using direct or indirect immunofluorescence: CD45 (555482; BD Biosciences), Lineage Cocktail 4 (i.e., CD2, CD3, CD4, CD7, CD8, CD10, CD11b, CD14, CD19, CD20, CD56, CD235a; BD Biosciences), and AC133/CD133 (130-080-801, Miltenyi Biotec). For direct labeling, cells were incubated with 1 μg/ml antibody for 30 min at room temperature. For indirect fluorescence, cells were first incubated with the primary antibody and then with 1 μg/ml secondary antibody for 45 min at room temperature. Cells were analyzed on a FACScan™ (BD Biosciences, Franklin, NJ, USA) using Cell Quest (BD Biosciences) and WinMDI (Scripps Research Institute, La Jolla, CA, USA) software. At least 5000 events were analyzed in each test. Mouse IgG1, IgG2a, and IgG2b (Becton Dickinson, San Jose, CA, USA) were used as isotype controls.

**Isolation, expansion, and purification of vselMSCs, and culture of unpurified MSCs (uMSCs) and ESCs**

Figure S1 shows the protocol of VSEL isolation and analysis. The vselMSCs were isolated and purified from isolated MNCs as previously described ^[2, 3]^. Briefly, MNCs were depleted of CD45^+^ Lineage^+^ (130-092-211) cells via micromagnetic bead selection (Miltenyi Biotec), cloned via limited dilution, and expanded in MSC complete medium (Iscove’s modified Dulbecco medium: IMDM with 20% fetal bovine serum, 2 mM L-glutamine, penicillin [100 U/ml] and streptomycin [100 µg/ml]) ^[4]^. Small and round cell clones were picked out and sorted by FACS using CD133–PE (130-080-801) and SH2-FITC (CD105, 130-098-774), and CD34-biotin (130-098-554) antibodies (Miltenyi Biotec). The gating protocol was tuned to include events that were consistent with the 5–7-µm size of the VSELs. CD34^–^CD133^+^SH2^+^ cells were re-suspended in ESC medium (Dulbecco’s modified Eagle’s medium [DMEM]/F12, Gibco Invitrogen) supplemented with 10% fetal calf serum (FCS), penicillin (100 units/ml), and streptomycin (100 μg/ml) ^[5]^, and plated at a density of 0.5 cells/well in 96-well, flat-bottomed culture plates. Based on Poisson statistics, the probability that clonal populations would derive from a single cell was ~95%. At 80% confluence, single-cloned cells were collected and defined as vselMSCs, and their purity and character were determined using FACS, morphology, differentiation, a gene chip, real-time RT-PCR, and western blotting. uMSCs were obtained from the abovementioned MNCs via the adherent culture method ^[2]^ (**Figure SI**).

The human embryonic stem cell (hESC) line H7 was purchased from SIDANSAI Biotechnology (Shanghai, China, 0204-001) and used as the positive control. Human ESCs were maintained at 37°C in 5% CO_2_ in hESC medium containing DMEM/F12 (Gibco), 20% knockout serum replacement (Invitrogen), 5 ng/ml human basic fibroblast growth factor (bFGF; Sigma-Aldrich, St. Louis, MO, USA), 100 μM nonessential amino acids (NEAA; Invitrogen), 100 μM β-mercaptoethanol (Sigma-Aldrich), 5% antibiotic/antimycotic (Invitrogen), and 2 μl/ml Fungizone (Invitrogen).

**FACS of vselMSCs**

Cells (2 × 10^5^/mL) were incubated in PBS with antibodies against CD34 (553733), CD44 (550989), CD71 (561938), CD147 (555962), SH2 (CD105, 560839), SH3 (CD73, 561254), stage-specific embryonic antigen-4 (SSEA-4, 560128) (BD Biosciences), and the abovementioned CD45, Lineage, and CD133. Mouse IgG1, IgG2a, and IgG2b (Becton Dickinson) were used as isotype controls, and marker expression was evaluated using a FACS apparatus as mentioned above. Cell viability was evaluated via the propidium iodide exclusion assay and by flow cytometry.

***In vitro* directed differentiation of vselMSCs**

Confluent vselMSC colonies were detached by incubation with 1 mg/ml collagenase (Invitrogen) for 30–60 min, and replated onto low-attachment 6-well plates (Fisher, Chino, CA, USA) in embryoid body (EB) medium consisting of DMEM-F12 (Invitrogen) supplemented with 15% defined fetal bovine serum (HyClone), 5% knockout serum replacement (Invitrogen), 1 mM L-glutamine (Invitrogen), 2 mM 2-mercaptoethanol, 0.1 mM NEAA (Invitrogen), and 1 mM penicillin/streptomycin (HyClone). For neural-directed differentiation, day 5 vselMSCs were plated on fibronectin (20 µg/ml)-coated dishes and cultured in DMEM/F12 supplemented with N2 and B27 (Invitrogen), 10 ng/ml bFGF, 1 ng/ml IGF, 1 ng/ml platelet-derived growth factor α polypeptide, and 10 ng/ml epidermal growth factor (all from PeproTech, Rocky Hill, NJ, USA) for an additional 5–7 days ^[6]^. For mesoderm differentiation ^[7]^, vselMSCs were cultured for 8 days in Stem Line II medium (Sigma-Aldrich) supplemented with 1× CD lipid concentrates, 2 mM GlutaMAX, 1× insulin transferrin selenium, penicillin/streptomycin (100 units/100 mg/ml) (all from Invitrogen), 400 µM monothioglycerol, and 50 mg/ml ascorbic acid (Sigma-Aldrich). The following growth factors were added: 10 ng/ml bone morphogenetic protein (BMP-4; R&D Systems, Minneapolis, MN, USA), 5 ng/ml bFGF (Invitrogen), and 20 ng/ml vascular endothelial growth factor (VEGF; R&D Systems). For ectoderm differentiation, vselMSCs were treated with 100 ng/ml activin (PeproTech) for 1 day and with 1% FBS and 100 ng/ml activin for the next 2 days in DMEM/F12 ^[8]^.

**Immunocytofluorescence**

Light images were collected on each cover slip under a light microscope (http://dict.baidu.com/). For immunocytofluorescence, cells were fixed with fresh 4% paraformaldehyde in PBS. Cells were incubated with primary antibodies at 1:100–500 dilution in 10% normal goat serum (NGS)/PBS overnight at 4°C. The primary antibodies used were rabbit anti–β-tubulin III (#5666, Cell Signaling Technology), mouse anti-glial fibrillary acidic protein (GFAP; #3760, Cell Signaling Technology), goat anti–troponin T (ab64623, Abcam), mouse anti-myosin heavy chain (MHC; NB300-284, Novus Biologicals), mouse anti–factor VIII (ab20837, Abcam), rabbit anti–alpha smooth muscle actin (α-SMA; ab5694), rabbit anti-human serum albumin (ab2406, Abcam), rabbit anti–alpha fetoprotein (AFP; #2137, Cell Signaling Technology), HIF-2α (NB100-902, Novus Biologicals), and Oct4 (NBP1-73963, Novus Biologicals). Secondary antibodies were species appropriate immunoglobulins linked to tetramethyl rhodamine isothiocyanate (TRITC) or fluorescein isothiocyanate (FITC) (Invitrogen). All cells were then stained with 4’,6-diamidino-2-phenylindole (DAPI, 10 µg/ml, Santa Cruz Biotechnology) in PBS for 5 min. All immunocytofluorescence experiments included control cells on cover slips processed in parallel with the omission of a primary antibody; no signal was observed in the red or blue channel in these preparations.

**Microarray analysis**

To analyze the anti-apoptotic genetic similarity between vselMSCs and hESCs, the cells were treated in a parallel culture with DMEM/F12. The treatment was performed over three sequential independent passages and hybridized to six Affymetrix HG-U133A chips. Total RNA was extracted using a High Pure RNA isolation kit (Roche Diagnostics, Mannheim, Germany) according to the manufacturer’s protocol. Array images were digitized by densitometric scanning on a Fluor-S MultiImager (Bio-Rad Laboratories, Hercules, CA, USA) and analyzed by using GEarray Expression Analysis Suite software (SuperArray). Values were normalized with respect to the signal for the housekeeping gene glyceraldehyde-3-phosphate dehydrogenase (GAPDH). Genes were considered differentially expressed if the ratio between the two cells was >1.5 ^[9]^.

**HIF-2α and Oct4 transfection**

pMXs retroviral plasmid vectors encoding HIF-2α or Oct4 expression (Addgene) were transfected with the viral packaging genes gag-pol (Addgene) into vselMSCs using Fugene HD reagent (Roche, Basel, Switzerland) as directed by the manufacturer’s instructions. HIF-2α or Oct4 siRNA and control siRNA duplexes (ON-TARGET plus, SMARTpool; Dharmacon) were transfected with pRL-TK plasmid vector (Promega, Madison, WI, USA) containing the *Renilla reniformis* luciferase gene into vselMSCs with Lipofectamine 2000 (Invitrogen) as described previously ^[10]^. Cells were collected 24 h after transfection for subsequent experiments.

**Hypoxic treatment**

Cells were removed and exposed to hypoxic (1%) oxygen levels in a water-jacketed CO_2_ incubator; the hypoxic oxygen level was maintained via regulated nitrogen injection (Forma Scientific). The hypoxic condition was maintained throughout the performance of all subsequent analyses.

**Cell viability, apoptosis, and proliferation analysis**

Cell proliferation and viability under normoxic and hypoxic conditions were determined using the trypan blue dye exclusion method or the CellTiter 96 AQueous One Solution Cell Proliferation Assay (MTS, Promega) following the company’s protocol. Apoptotic cell death under normoxic and hypoxic conditions was evaluated through annexin V (Roche Diagnostic, Indianapolis, IN) and propidium iodide (PI) staining using a FACScan flow cytometer and Cell Quest software (Becton Dickinson Immunocytometry Systems).

For cell proliferation assays, cells were immunostained with anti-Ki67 antibody following the company’s protocol. The cells (2 × 10^5^/ml) were then incubated with 4 ml antibodies against Ki67 (ab15580, Abcam). Proliferation was considered the proportion of cells that expressed both green fluorescent protein (GFP) and Ki67 relative to the GFP-positive cells.

**GFP labeling**

At 24 h after transfection with the HIF-2α,Oct4, siHIF-2α, siOct4, or control siRNA vectors, cells were co-transfected with a lentiviral vector containing enhanced GFP cDNA, as described previously ^[8]^. More than 70% of vselMSCs were GFP-positive, as determined by flow cytometry.

**MI model and treatment**

The Animal Care and Use Committee of Southern Medical University approved all procedures involving animals, which were in compliance with the NIH Guide for the Care and Use of Laboratory Animals (the updated [2011] version of the NIH guidelines). Under general anesthesia with a mixture of ketamine/xylazine (100/15 mg/kg, intraperitoneal [i.p.]), MI was induced in male Sprague-Dawley rats (200–250 g) obtained from the Tongji Animal Administration Center by ligating the left anterior descending coronary artery as previously described ^[2]^.

The adequacy of the anesthesia was monitored by the level and stability of the mean arterial pressure (MAP) and absence of corneal reflex, and adequate levels of anesthesia and analgesia were ensured with supplemental i.p. injection of pentobarbital sodium given as required. The animals were then randomized to receive saline injection or cell therapy. The total volume of saline or cell suspension injected was ~100 µl, and each animal in the cell treatment groups received a total of 5 × 10^6^ cells. Injections were administered to the infarct and peri-infarct regions at four sites separated by 1–2 cm. Therefore, all animals were divided into 11 groups: sham operation (SHAM), PBS (PBS), ^WT^uMSCs (^WT^uM), ^WT^vselMSCs,^HIF-2α+^vselMSCs group (HIF-2α^+^), ^Oct4^vselMSCs (Oct4^+^), ^siHIF-2α+^vselMSCs (siHIF-2α^+^), and ^siOct4+^vselMSCs (siOct4^+^), receiving vselMSCs treated with HIF-2α and Oct4 co-transfection (HIF-2α^+^Oct4^+^), receiving vselMSCs overexpressing HIF-2α with Oct4 silencing (HIF-2α^+^siOct4^+^), and receiving vselMSCs overexpressing Oct4 with HIF-2α silencing (siHIF-2α^+^Oct4^+^), respectively. Fifteen animals were studied in each subgroup. To minimize postoperative pain, 2.5% bupivacaine was sprayed at the point of incision immediately before closure, and buprenorphine hydrochloride (0.03 mg/kg) was administered intramuscularly. After the final layer of skin was closed, triple antibiotic ointment (neomycin sulfate, polymyxin B sulfate, and bacitracin zinc) was applied to the wound. Cyclosporin A (Novartis Pharma) was administered daily (5 mg/kg, i.h.) from the first day after MI until the animals were sacrificed on day 30.

**Echocardiography**

Under general anesthesia as described above, echocardiography was performed on a 7.5-MHz phased array transducer (Acuson Sequoia 256) ^[2]^ by an experienced technician who was blinded to treatment group identity; two-dimensional (2D) images were obtained at the mid-papillary and apical levels. Left ventricular end diastolic volume (LVEDV), internal diameter (LVEDd), anterior wall thickness (LVAWd), and posterior wall thickness at diastolic phase (LVPWd) were measured using the biplane area–length method. LV fractional shortening (FS) was calculated according to the modified Simpson method: FS (%) = [(LVIDd - LVIDs)/LVIDd] × 100, where LVID is LV internal dimension, s is systole, and d is diastole. Infarct size was assessed at the end of the study by planimetry on 2D short axis–acquired real-time images of the LV according to a previous described procedure ^[11]^.All measurements were averaged for three consecutive cardiac cycles.

**Real-time RT-PCR**

Total RNA was isolated from cultured cells or homogenized heart tissue using TRIzol reagent (Gibco BRL). The RNA was reverse-transcribed with a TaqMan cDNA Synthesis Kit (Applied Biosystems, Foster City, CA, USA) and amplified on a TaqMan 7500 (Applied Biosystems). Primer and probe sequences are listed in Table SI. Expression was calculated via the comparative cycle threshold (CT) method and normalized to that of GAPDH, which was included as an internal control; thresholds for the individual reactions were determined by using ABI Prism SDS 2.0 data processing software (Applied Biosystems).

**Western blotting**

Cell lysates and myocardial tissues were prepared as described previously ^[2]^, and 100 mg total protein per lane was loaded on the gel. Assessments were performed with primary antibodies against Oct4 (NBP1-73963, Novus Biologicals), Sox2 (AB5603, Chemicon), Nanog (AF1997, R&D Systems), Klf4 (#4083, Cell Signaling Technology), HIF-2 (NB100-902, Novus Biologicals), VEGF (sc-152, Santa Cruz Biotechnology), survivin (MA5-11680, Thermo Scientific), caspase-3 (#9662, Cell Signaling Technology), MHC (NB300-284, Novus Biologicals), troponin T (bs-2804R, Bioss Inc.), and factor VIII (sc-27647, Santa Cruz Biotechnology). GAPDH (1:300, MAB374, Chemicon) was detected as a protein control, and a chemiluminescence detection kit (Amersham Biosciences) was used to detect the secondary antibody.

**Histology, immunohistochemistry, and immunofluorescence**

The rats were killed with i.p. injection of overdose barbiturate (150 mg/kg) after the echocardiography examinations, weighed, and their hearts were removed. Five hearts were randomly selected for subsequent cell collection. MI size was evaluated by 2,3,5-triphenyltetrazolium chloride triazole (TTC) staining ^[12]^. The left ventricles were divided into three equal parts from the apex to the base, and the middle portion was immersed in 0.09 mol/l PBS (pH 7.4) containing 1.0% TTC (Sigma-Aldrich) for 20 min at 37°C. The size of the infarct was evaluated using a digital imaging program (Scion ImageJ) and expressed as a percentage of the area of the entire LV area.

The peri-infarct regions were embedded in paraffin or frozen for cryostat sectioning and were then stained by immunohistochemistry or immunofluorescence. The cryostat sections were stained with antibodies against the following proteins: Ang-1, bFGF, VEGF, Bcl2, Survivin, and Caspase 3. Secondary antibodies were species appropriate immunoglobulins linked to tetramethyl rhodamine isothiocyanate (TRITC) (Invitrogen). All cells were then stained with 4’,6-diamidino-2-phenylindole (DAPI, 10 µg/ml, Santa Cruz Biotechnology) in PBS for 5 min.

The paraffin sections were stained with anti-factor VIII antibody, and counterstained with an immunoperoxidase kit (Vector Labs, Burlingame, CA, USA). Vessels were identified as round or elliptical structures with a central lumen lined by factor VIII^+^ cells and counted in 10 randomly selected fields per section, 10 sections per heart, by a pathologist who was blinded to treatment group identity. Vascularity was expressed as the number of factor VIII^+^ vessels per square millimeter ^[2]^.

Apoptotic signaling was evaluated by staining sections with primary antibodies against survivin, Bcl-2, and caspase-3; primary antibody staining was visualized using TRITC-conjugated goat anti-mouse IgG and TRITC-conjugated goat anti-rabbit IgG secondary antibodies (Jackson ImmunoResearch Laboratories, Inc., PA, USA).

**Engraftment, proliferation, and cardiomyocyte differentiation**

Cells were collected from the left ventricles of five randomly selected hearts per experimental group as previously described ^[13]^. Briefly, the heart was excised and perfused retrograde with Ca^2+^-free perfusion buffer. The left ventricle was minced in collagenase, and the solution was filtered through a nylon mesh. The collected cells were washed with PBS and analyzed with a flow cytometry apparatus (Becton Dickinson, Mountain View, CA, USA). Engraftment was evaluated by determining the proportion of cells that expressed EGFP relative to all isolated ventricular cells. The EGFP-positive cells were isolated by FACS. Then, the cells (2 × 10^5^/ml) were then incubated with 4 ml antibodies against Ki67, factor VIII, or MHC (ab15, Abcam). Proliferation was evaluated by calculating the proportion of cells that expressed both GFP and Ki67, and vascular/cardiomyocyte differentiation was evaluated by calculating the proportion of cells that expressed both GFP and factor VIII/MHC relative to all GFP-positive cells. EGFP-positive cells isolated from the region surrounding the infarct site were also performed to analyze expression of the myocardiocyte marker MHC and blood vascular marker factor VIII by immunocytofluorescence as above-mentioned.

**Statistical analysis**

The results are expressed as the mean ± standard error of the mean (SEM) and tested for significance using analysis of variance (ANOVA) for multiple comparisons. Chi-square analysis was used to compare survival rates between groups. A p-value of <0.05 was considered statistically significant.

**References**

1. [Ratajczak MZ](http://www.ncbi.nlm.nih.gov/pubmed?term=Ratajczak MZ%5BAuthor%5D&cauthor=true&cauthor_uid=22498452), [Shin DM](http://www.ncbi.nlm.nih.gov/pubmed?term=Shin DM%5BAuthor%5D&cauthor=true&cauthor_uid=22498452), [Liu R](http://www.ncbi.nlm.nih.gov/pubmed?term=Liu R%5BAuthor%5D&cauthor=true&cauthor_uid=22498452), [Mierzejewska K](http://www.ncbi.nlm.nih.gov/pubmed?term=Mierzejewska K%5BAuthor%5D&cauthor=true&cauthor_uid=22498452), [Ratajczak J](http://www.ncbi.nlm.nih.gov/pubmed?term=Ratajczak J%5BAuthor%5D&cauthor=true&cauthor_uid=22498452), [Kucia M](http://www.ncbi.nlm.nih.gov/pubmed?term=Kucia M%5BAuthor%5D&cauthor=true&cauthor_uid=22498452), [Zuba-Surma EK](http://www.ncbi.nlm.nih.gov/pubmed?term=Zuba-Surma EK%5BAuthor%5D&cauthor=true&cauthor_uid=22498452). Very small embryonic/epiblast-like stem cells (VSELs) and their potential role in aging and organ rejuvenation--an update and comparison to other primitive small stem cells isolated from adult tissues. Aging (Albany NY) 2012; 4(4): 235-246.
2. Zhang S, Ge J, Sun A, Xu D, Qian J, Lin J, Zhao Y, Hu H, Li Y, Wang K, Zou Y. [Comparison of various kinds of bone marrow stem cells for the repair of infarcted myocardium: single clonally purified non-hematopoietic mesenchymal stem cells serve as a superior source.](http://www.ncbi.nlm.nih.gov/pubmed/16795039?ordinalpos=11&itool=EntrezSystem2.PEntrez.Pubmed.Pubmed_ResultsPanel.Pubmed_DefaultReportPanel.Pubmed_RVDocSum) J Cell Biochem 2006; 99: 1132-1147.
3. [Wojakowski](http://www.ncbi.nlm.nih.gov/pubmed/?term=Wojakowski W%5Bauth%5D) W, Kucia M, [Liu](http://www.ncbi.nlm.nih.gov/pubmed/?term=Liu R%5Bauth%5D) R, [Zuba-Surma](http://www.ncbi.nlm.nih.gov/pubmed/?term=Zuba-Surma E%5Bauth%5D) E, [Jadczyk](http://www.ncbi.nlm.nih.gov/pubmed/?term=Jadczyk T%5Bauth%5D) T, [Bachowski](http://www.ncbi.nlm.nih.gov/pubmed/?term=Bachowski R%5Bauth%5D) R, [Nabiałek](http://www.ncbi.nlm.nih.gov/pubmed/?term=Nabia%26%23x00142%3Bek E%5Bauth%5D) E, [Kaźmierski](http://www.ncbi.nlm.nih.gov/pubmed/?term=Ka%26%23x0017a%3Bmierski M%5Bauth%5D) M, [Ratajczak](http://www.ncbi.nlm.nih.gov/pubmed/?term=Ratajczak MZ%5Bauth%5D) MZ, [Tendera](http://www.ncbi.nlm.nih.gov/pubmed/?term=Tendera M%5Bauth%5D) M. Circulating very small embryonic-like stem cells in cardiovascular disease. J Cardiovasc Transl Res 2011; 4: 138–144.
4. [De Bari C](http://www.ncbi.nlm.nih.gov/pubmed?term="De Bari C"%5BAuthor%5D), [Dell'Accio F](http://www.ncbi.nlm.nih.gov/pubmed?term="Dell'Accio F"%5BAuthor%5D), [Vanlauwe J](http://www.ncbi.nlm.nih.gov/pubmed?term="Vanlauwe J"%5BAuthor%5D), [Eyckmans J](http://www.ncbi.nlm.nih.gov/pubmed?term="Eyckmans J"%5BAuthor%5D), [Khan IM](http://www.ncbi.nlm.nih.gov/pubmed?term="Khan IM"%5BAuthor%5D), [Archer CW](http://www.ncbi.nlm.nih.gov/pubmed?term="Archer CW"%5BAuthor%5D), [Jones EA](http://www.ncbi.nlm.nih.gov/pubmed?term="Jones EA"%5BAuthor%5D), [McGonagle D](http://www.ncbi.nlm.nih.gov/pubmed?term="McGonagle D"%5BAuthor%5D), [Mitsiadis TA](http://www.ncbi.nlm.nih.gov/pubmed?term="Mitsiadis TA"%5BAuthor%5D), [Pitzalis C](http://www.ncbi.nlm.nih.gov/pubmed?term="Pitzalis C"%5BAuthor%5D), [Luyten FP](http://www.ncbi.nlm.nih.gov/pubmed?term="Luyten FP"%5BAuthor%5D). Mesenchymal multipotency of adult human periosteal cells demonstrated by single-cell lineage analysis. [Arthritis Rheum](http://www.ncbi.nlm.nih.gov/pubmed/16575900#%23) 2006; 54: 1209-1221.
5. Claros S, Rodríguez-Losada N, Cruz E, Guerado E, Becerra J, Andrades JA.[Characterization of adult stem/progenitor cell populations from bone marrow in a three-dimensional collagen gel culture system.](http://www.ncbi.nlm.nih.gov/pubmed/22472743)Cell Transplant2012;21:2021- 2032.
6. Carpenter MK, Inokuma MS, Denham J, Mujtaba T, Chiu CP, Rao MS. Enrichment of neurons and neural precursors from human embryonic stem cells. Exp Neurol 2001; 172(2): 383-397.
7. [Dravid G](http://www.ncbi.nlm.nih.gov/pubmed?term=Dravid G%5BAuthor%5D&cauthor=true&cauthor_uid=21179006), [Zhu Y](http://www.ncbi.nlm.nih.gov/pubmed?term=Zhu Y%5BAuthor%5D&cauthor=true&cauthor_uid=21179006), [Scholes J](http://www.ncbi.nlm.nih.gov/pubmed?term=Scholes J%5BAuthor%5D&cauthor=true&cauthor_uid=21179006), [Evseenko D](http://www.ncbi.nlm.nih.gov/pubmed?term=Evseenko D%5BAuthor%5D&cauthor=true&cauthor_uid=21179006), [Crooks GM](http://www.ncbi.nlm.nih.gov/pubmed?term=Crooks GM%5BAuthor%5D&cauthor=true&cauthor_uid=21179006).Dysregulated gene expression during hematopoietic differentiation from human embryonic stem cells.Mol Ther2011;19(4):768-781.
8. [Alva JA](http://www.ncbi.nlm.nih.gov/pubmed?term=Alva JA%5BAuthor%5D&cauthor=true&cauthor_uid=21948699), [Lee GE](http://www.ncbi.nlm.nih.gov/pubmed?term=Lee GE%5BAuthor%5D&cauthor=true&cauthor_uid=21948699), [Escobar EE](http://www.ncbi.nlm.nih.gov/pubmed?term=Escobar EE%5BAuthor%5D&cauthor=true&cauthor_uid=21948699), [Pyle AD](http://www.ncbi.nlm.nih.gov/pubmed?term=Pyle AD%5BAuthor%5D&cauthor=true&cauthor_uid=21948699). Phosphatase and tensin homolog regulates the pluripotent state and lineage fate choice in human embryonic stem cells. Stem Cells 2011; 29(12): 1952-1962.
9. [Maillard CM](http://www.ncbi.nlm.nih.gov/pubmed?term=Maillard CM%5BAuthor%5D&cauthor=true&cauthor_uid=18753414), [Bouquet C](http://www.ncbi.nlm.nih.gov/pubmed?term=Bouquet C%5BAuthor%5D&cauthor=true&cauthor_uid=18753414), [Petitjean MM](http://www.ncbi.nlm.nih.gov/pubmed?term=Petitjean MM%5BAuthor%5D&cauthor=true&cauthor_uid=18753414), [Mestdagt M](http://www.ncbi.nlm.nih.gov/pubmed?term=Mestdagt M%5BAuthor%5D&cauthor=true&cauthor_uid=18753414), [Frau E](http://www.ncbi.nlm.nih.gov/pubmed?term=Frau E%5BAuthor%5D&cauthor=true&cauthor_uid=18753414), [Jost M](http://www.ncbi.nlm.nih.gov/pubmed?term=Jost M%5BAuthor%5D&cauthor=true&cauthor_uid=18753414), [Masset AM](http://www.ncbi.nlm.nih.gov/pubmed?term=Masset AM%5BAuthor%5D&cauthor=true&cauthor_uid=18753414), [Opolon PH](http://www.ncbi.nlm.nih.gov/pubmed?term=Opolon PH%5BAuthor%5D&cauthor=true&cauthor_uid=18753414), [Beermann F](http://www.ncbi.nlm.nih.gov/pubmed?term=Beermann F%5BAuthor%5D&cauthor=true&cauthor_uid=18753414), [Abitbol MM](http://www.ncbi.nlm.nih.gov/pubmed?term=Abitbol MM%5BAuthor%5D&cauthor=true&cauthor_uid=18753414), [Foidart JM](http://www.ncbi.nlm.nih.gov/pubmed?term=Foidart JM%5BAuthor%5D&cauthor=true&cauthor_uid=18753414), [Perricaudet MJ](http://www.ncbi.nlm.nih.gov/pubmed?term=Perricaudet MJ%5BAuthor%5D&cauthor=true&cauthor_uid=18753414), [Noël AC](http://www.ncbi.nlm.nih.gov/pubmed?term=No?l AC%5BAuthor%5D&cauthor=true&cauthor_uid=18753414). Reduction of brain metastases in plasminogen activator inhibitor-1-deficient mice with transgenic ocular tumors. Carcinogenesis 2008; 29(11): 2236-2242.
10. [Schultz K](http://www.ncbi.nlm.nih.gov/pubmed?term=Schultz K%5BAuthor%5D&cauthor=true&cauthor_uid=16399861), [Fanburg BL](http://www.ncbi.nlm.nih.gov/pubmed?term=Fanburg BL%5BAuthor%5D&cauthor=true&cauthor_uid=16399861), [Beasley D](http://www.ncbi.nlm.nih.gov/pubmed?term=Beasley D%5BAuthor%5D&cauthor=true&cauthor_uid=16399861). Hypoxia and hypoxia-inducible factor-1alpha promote growth factor-induced proliferation of human vascular smooth muscle cells. Am J Physiol Heart Circ Physiol 2006; 290: H2528-H2534.
11. [Ciulla MM](http://www.ncbi.nlm.nih.gov/pubmed/?term=Ciulla MM%5BAuthor%5D&cauthor=true&cauthor_uid=18549470), [Montelatici E](http://www.ncbi.nlm.nih.gov/pubmed/?term=Montelatici E%5BAuthor%5D&cauthor=true&cauthor_uid=18549470), [Ferrero S](http://www.ncbi.nlm.nih.gov/pubmed/?term=Ferrero S%5BAuthor%5D&cauthor=true&cauthor_uid=18549470), [Braidotti P](http://www.ncbi.nlm.nih.gov/pubmed/?term=Braidotti P%5BAuthor%5D&cauthor=true&cauthor_uid=18549470), [Paliotti R](http://www.ncbi.nlm.nih.gov/pubmed/?term=Paliotti R%5BAuthor%5D&cauthor=true&cauthor_uid=18549470), [Annoni G](http://www.ncbi.nlm.nih.gov/pubmed/?term=Annoni G%5BAuthor%5D&cauthor=true&cauthor_uid=18549470), [De Camilli E](http://www.ncbi.nlm.nih.gov/pubmed/?term=De Camilli E%5BAuthor%5D&cauthor=true&cauthor_uid=18549470), [Busca G](http://www.ncbi.nlm.nih.gov/pubmed/?term=Busca G%5BAuthor%5D&cauthor=true&cauthor_uid=18549470), [Chiappa L](http://www.ncbi.nlm.nih.gov/pubmed/?term=Chiappa L%5BAuthor%5D&cauthor=true&cauthor_uid=18549470), [Rebulla P](http://www.ncbi.nlm.nih.gov/pubmed/?term=Rebulla P%5BAuthor%5D&cauthor=true&cauthor_uid=18549470), [Magrini F](http://www.ncbi.nlm.nih.gov/pubmed/?term=Magrini F%5BAuthor%5D&cauthor=true&cauthor_uid=18549470), [Lazzari L](http://www.ncbi.nlm.nih.gov/pubmed/?term=Lazzari L%5BAuthor%5D&cauthor=true&cauthor_uid=18549470).Potential advantages of cell administration on the inflammatory response compared to standard ACE inhibitortreatment in experimental myocardial infarction.[J Transl Med](http://www.ncbi.nlm.nih.gov/pubmed/?term=Potential+advantages+of+cell+administration+on+the+inflammatory+response+compared+to+standard+ACE+inhibitor+treatment+in+experimental+myocardial+infarction) 2008;6:30.
12. [Lin D](http://www.ncbi.nlm.nih.gov/pubmed/?term=Lin D%5BAuthor%5D&cauthor=true&cauthor_uid=26632817), [Ma J](http://www.ncbi.nlm.nih.gov/pubmed/?term=Ma J%5BAuthor%5D&cauthor=true&cauthor_uid=26632817), [Xue Y](http://www.ncbi.nlm.nih.gov/pubmed/?term=Xue Y%5BAuthor%5D&cauthor=true&cauthor_uid=26632817), [Wang Z](http://www.ncbi.nlm.nih.gov/pubmed/?term=Wang Z%5BAuthor%5D&cauthor=true&cauthor_uid=26632817).Penehyclidine Hydrochloride Preconditioning Provides Cardioprotection in a Rat Model of MyocardialIschemia/Reperfusion Injury.[PLoS One](http://www.ncbi.nlm.nih.gov/pubmed/26632817) 2015;10(12):e0138051.
13. [Tabe Y](http://www.ncbi.nlm.nih.gov/pubmed?term=Tabe Y%5BAuthor%5D&cauthor=true&cauthor_uid=24505547), [Shi YX](http://www.ncbi.nlm.nih.gov/pubmed?term=Shi YX%5BAuthor%5D&cauthor=true&cauthor_uid=24505547), [Zeng Z](http://www.ncbi.nlm.nih.gov/pubmed?term=Zeng Z%5BAuthor%5D&cauthor=true&cauthor_uid=24505547), [Jin L](http://www.ncbi.nlm.nih.gov/pubmed?term=Jin L%5BAuthor%5D&cauthor=true&cauthor_uid=24505547), [Shikami M](http://www.ncbi.nlm.nih.gov/pubmed?term=Shikami M%5BAuthor%5D&cauthor=true&cauthor_uid=24505547), [Hatanaka Y](http://www.ncbi.nlm.nih.gov/pubmed?term=Hatanaka Y%5BAuthor%5D&cauthor=true&cauthor_uid=24505547), [Miida T](http://www.ncbi.nlm.nih.gov/pubmed?term=Miida T%5BAuthor%5D&cauthor=true&cauthor_uid=24505547), [Hsu FJ](http://www.ncbi.nlm.nih.gov/pubmed?term=Hsu FJ%5BAuthor%5D&cauthor=true&cauthor_uid=24505547), [Andreeff M](http://www.ncbi.nlm.nih.gov/pubmed?term=Andreeff M%5BAuthor%5D&cauthor=true&cauthor_uid=24505547), [Konopleva M](http://www.ncbi.nlm.nih.gov/pubmed?term=Konopleva M%5BAuthor%5D&cauthor=true&cauthor_uid=24505547). TGF-β-Neutralizing Antibody 1D11 Enhances Cytarabine-Induced Apoptosis in AML Cells in the Bone Marrow Microenvironment. PLoS One 2013; 8(6): e62785.

**Figure legend**

**Figure S1**: **The experimental flow of vselMSC development and analysis of the cooperation between HIF-2α and Oct4 in regulating vselMSC pluripotency, survival, proliferation, and therapeutic potential***.* Blood was collected from the affected coronary artery, and MNCs were isolated from the samples using erythrocyte lysis buffer. MNCs were depleted of CD45^+^ Lineage^+^ cells via micromagnetic bead selection, cloned via limited-dilution culture, and expanded in MSC complete medium and ESC medium. Cells were stained for SH2, CD133, and CD34 expression, and CD34^–^CD133^+^SH2^+^ cells were collected via flow cytometry; the gating protocol was tuned to include events that were consistent with the 5–7-µm size of vselMSCs. uMSCs were isolated using a standard adherent culture protocol.
